# Impaired Th17 polarization of phenotypically naive CD4^+^ T-cells during chronic HIV-1 infection and potential restoration with early ART

**DOI:** 10.1186/s12977-015-0164-6

**Published:** 2015-04-30

**Authors:** Sandrina DaFonseca, Julia Niessl, Sylvia Pouvreau, Vanessa Sue Wacleche, Annie Gosselin, Aurélie Cleret-Buhot, Nicole Bernard, Cécile Tremblay, Mohammad-Ali Jenabian, Jean-Pierre Routy, Petronela Ancuta

**Affiliations:** Faculty of Medicine, Department of Microbiology, Infectiology and Immunology, Université de Montréal, Montreal, QC Canada; CHUM-Research Centre, 900 rue Saint-Denis, Tour Viger R, room R09.416, Montreal, QC H2X 0A9 Canada; Chronic Viral Illness Service, McGill University Health Centre, Montreal, QC Canada; Research Institute of the McGill University Health Centre, Montreal, QC Canada; Division of Experimental Medicine, McGill University, Montreal, QC Canada; Division of Clinical Immunology, McGill University Health Centre, Montreal, QC Canada; Division of Hematology, McGill University Health Centre, Montreal, QC Canada; Département des sciences Biologiques, Université du Québec à Montréal, Montreal, QC Canada

**Keywords:** HIV, Th17 cells, Regulatory T cells, CD25, CD127, Antiretroviral therapy

## Abstract

**Background:**

Depletion of mucosal Th17 cells during HIV/SIV infections is a major cause for microbial translocation, chronic immune activation, and disease progression. Mechanisms contributing to Th17 deficit are not fully elucidated. Here we investigated alterations in the Th17 polarization potential of naive-like CD4^+^ T-cells, depletion of Th17-commited subsets during HIV pathogenesis, and Th17 restoration in response to antiretroviral therapy (ART).

**Results:**

Peripheral blood CD4^+^ T-cells expressing a naive-like phenotype (CD45RA^+^CCR7^+^) from chronically HIV-infected subjects receiving ART (CI on ART; median CD4 counts 592 cells/μl; viral load: <50 HIV-RNA copies/ml; time since infection: 156 months) compared to uninfected controls (HIV-) were impaired in their survival and Th17 polarization potential *in vitro*. In HIV- controls, IL-17A-producing cells mainly originated from naive-like T-cells with a regulatory phenotype (nTregs: CD25^high^CD127^−^FoxP3^+^) and from CD25^+^CD127^+^FoxP3^−^ cells (DP, double positive). Th17-polarized conventional naive CD4^+^ T-cells (nT: CD25^−^CD127^+^FoxP3^−^) also produced IL17A, but at lower frequency compared to nTregs and DP. In CI on ART subjects, the frequency/counts of nTreg and DP were significantly diminished compared to HIV- controls, and this paucity was further associated with decreased proportions of memory T-cells producing IL-17A and expressing Th17 markers (CCR6^+^CD26^+^CD161^+^, mTh17). nTregs and DP compared to nT cells harbored superior levels of integrated/non-integrated HIV-DNA in CI on ART subjects, suggesting that permissiveness to integrative/abortive infection contributes to impaired survival and Th17 polarization of lineage-committed cells. A cross-sectional study in CI on ART subjects revealed that nTregs, DP and mTh17 counts were negatively correlated with the time post-infection ART was initiated and positively correlated with nadir CD4 counts. Finally, a longitudinal analysis in a HIV primary infection cohort demonstrated a tendency for increased nTreg, DP, and mTh17 counts with ART initiation during the first year of infection.

**Conclusions:**

These results support a model in which the paucity of phenotypically naive nTregs and DP cells, caused by integrative/abortive HIV infection and/or other mechanisms, contributes to Th17 deficiency in HIV-infected subjects. Early ART initiation, treatment intensification with integrase inhibitors, and/or other alternative interventions aimed at preserving/restoring the pool of cells prone to acquire Th17 functions may significantly improve mucosal immunity in HIV-infected subjects.

**Electronic supplementary material:**

The online version of this article (doi:10.1186/s12977-015-0164-6) contains supplementary material, which is available to authorized users.

## Background

HIV/SIV infections are associated with a massive depletion of CD4^+^ T-cells from the gut-associated lymphoid tissues (GALT), together with intestinal histological abnormalities characterized by epithelial cell apoptosis and impaired mucosal barrier integrity [1-3]. Among CD4^+^ T-cells, Th17 cells are preferentially depleted from the GALT of HIV-infected individuals with rapid disease progression [4,5]. Th17 cell depletion from the GALT of HIV-infected subjects and SIV-infected rhesus macaques is considered to be a major cause for microbial translocation, chronic immune activation, and disease progression [3,6-13]. The restoration of CD4^+^ T-cells in the GALT of HIV-infected subjects receiving viral suppressive antiretroviral therapy (ART) is associated with an enhanced frequency of Th17 cells and polyfunctional HIV-specific T-cell responses [5,14,15]. However, the quantitative and qualitative restoration of Th17 cells under long-term ART is only partial in chronically infected subjects [5,8,14]. Very recent studies demonstrated that ART initiation during the early acute phases of HIV infection (Fiebig I-II), but not during the late acute and chronic phases, permits the preservation of Th17 cell numbers/functions at mucosal level [16]. The challenges of early HIV diagnosis render immediate ART initiation almost utopic even in high income countries.

Considering the critical role played by Th17 cells in mucosal homeostasis [17-20] and HIV disease progression [16], understanding mechanisms of Th17 alterations during HIV/SIV infections continues to be the focus of active investigations. Studies by our group and others demonstrated that memory Th17 cells are highly permissive to HIV infection *in vitro* and *in vivo* [8,21-25] thus, implying a deleterious role of HIV infection *per se* on Th17 cell survival. Other documented mechanisms underlying Th17 deficiency during HIV/SIV infections include *(i)* altered trafficking potential of memory Th17 cells into mucosal sites [26,27]; *(ii)* increased ratios between regulatory T-cells *versus* Th17 cells at mucosal level due to enhanced indoleamine 2,3-dioxygenase 1 (IDO)-mediated tryptophan catabolism by mucosal dendritic cells (DC) [28,29]; and/or *(iii)* depletion of mucosal CD103^+^ DC [30], a subset involved in Th17 differentiation [31,32]. The Th17 polarization of naive T-cells requires specific signals *via* cytokines such as TGF-β, IL-6, IL-1β, and IL-23 [33-35]. Levels of TGF-β [36], IL-6 [37], and IL-1 [38] are documented to be upregulated during the course of HIV-infection. IL-23 levels are upregulated during HIV primary infection [39], but whether IL-23 production is altered during the chronic phase of infection requires further investigations [40,41]. One cytokine that appears to be limiting is IL-21, a cytokine discovered to be involved in an alternative Th17 differentiation pathway [42-44]. Our group reported a deficit in IL-21 expression associated with HIV infection, deficit that was partially restored by ART [45,46]. Decreased IL-21 levels were also reported during SIV infection [47] and the administration of recombinant IL-21 led to the restoration/preservation of Th17 responses at mucosal level in SIV-infected rhesus macaques [12]. Finally, the over expression of negative regulators implicated in the inhibition of Th17 differentiation was linked to Th17 deficiency in a SIV model of infection [48]. Together, these advances reflect the complex and not fully elucidated mechanisms underlying Th17 alterations during HIV/SIV infections.

A fraction of human peripheral blood CD4^+^ T-cells expressing the naive markers CD45RA and CCR7 [49] and a regulatory phenotype (nTregs: CD25^high^CD127^−^FoxP3^+^) preferentially acquire Th17 features *in vitro* [35,50]. The concept that nTregs include Th17-lineage committed cells is consistent with the well documented differentiation relationship between Th17 and Tregs [51,52] and in line with the identification of suppressive Tregs that express IL-17 (IL-17^+^ Tregs) [53]. The common origin of Tregs and Th17 cells is further supported by very recent studies in humans demonstrating the differentiation of IL-17-producing effector and regulatory T-cells from phenotypically naive (CD45RO^−^) CCR6^+^FoxP3^+^Helios^−^ CD4^+^ T-cells [54,55]. Whether Th17 deficiency in HIV-infected subjects is associated with the paucity of Th17-lineage committed precursors remains unknown.

In this study, we investigated alterations in the Th17 polarization potential of phenotypically naive CD4^+^ T-cells, sought to identify specific naive-like Th17-commited T-cell subsets that are depleted during HIV pathogenesis, and assessed the restoration of these subsets in response to antiretroviral therapy (ART). Studies were performed using peripheral blood samples collected from recently HIV-infected untreated (RI) and chronically infected aviremics under ART (CI on ART), as well as longitudinal samples from HIV-infected subjects with ART administered during the first year of infection. Our results support a model in which the paucity of phenotypically naive CD4^+^ T-cell subsets enriched in Th17-lineage committed cells represents a new mechanism contributing to Th17 deficiency in chronically HIV-infected subjects receiving ART. New therapeutic strategies such as early ART initiation and treatment intensification with integrase inhibitors are needed for the preservation of Th17 precursors and an optimal restoration of mucosal immunity in HIV-infected subjects.

## Results

### Phenotypically naive CD4^+^ T-cells from HIV-infected subjects are impaired in their Th17 polarization potential *in vitro*

**Figure 1:**
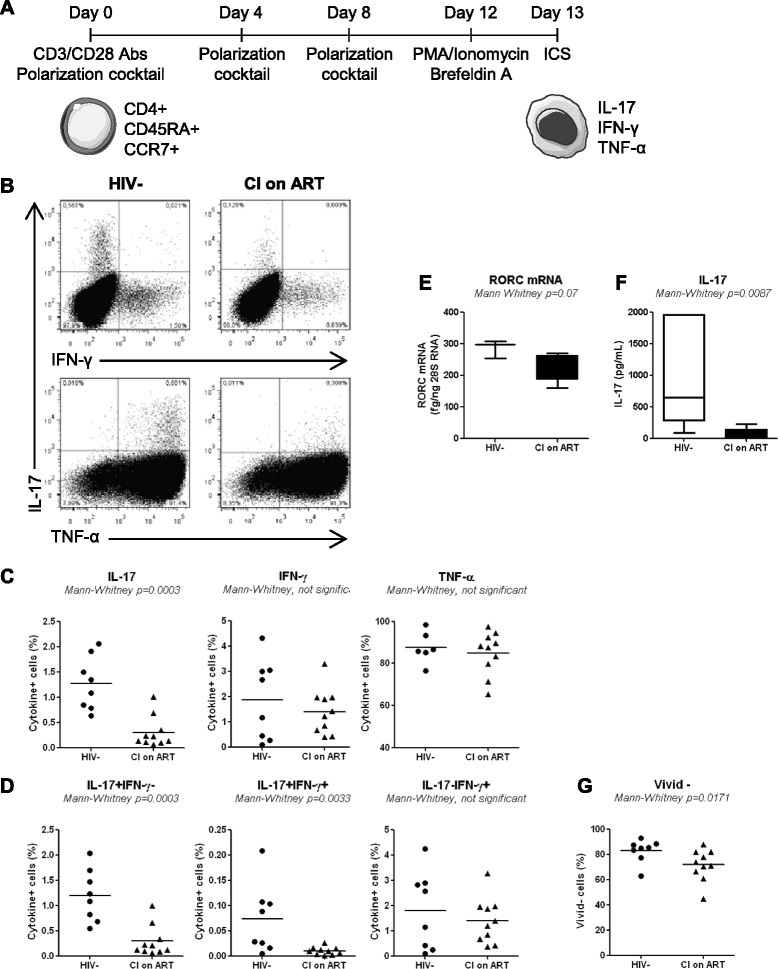
Phenotypically naive CD4^+^ T-cells from CI on ART subjects are impaired in their Th17-polarization potential *in vitro*. Total CD4^+^ T-cells were isolated by negative selection using magnetic beads (Miltenyi) and stained with a cocktail of CD8, CD19, CD56, CD45RA, and CCR7 Abs and the viability dye Vivid. **(A)** Shown is the schematic experimental design. Briefly, naive-like CD4^+^ T-cells (CD45RA^+^CCR7^+^ phenotype) lacking CD8, CD19, and CD56 expression, were sorted by flow cytometry (as in Additional file 1: Figure S1) and stimulated *via* CD3/CD28 under Th17 polarizing conditions for 12 days. Media containing polarizing cytokines and Abs together with IL-2 was refreshed at day 4 and 8 post-culture. At day 12, cells were stimulated with PMA and Ionomycin in the presence of Brefeldin A for 17 hours. Cells were analyzed by flow cytometry for cytokine expression upon intracellular staining with specific Abs. Vivid-positive cells were excluded from the analysis. **(B)** Shown is the frequency of cells expressing intracellular IL-17A, IFN-γ, and/or TNF-α in representative HIV- control and CI on ART subject. **(C-D)** Shown are statistical analysis of single cytokine expression **(C)** and cytokine co-expression **(D)** in Th17-polarized cells from HIV- controls (n = 8) and CI on ART (n = 10) subjects. **(E-F)** At day 8 of polarization cell pellets and culture supernatants were harvested for the quantification of RORC mRNA (n = 3 HIV- and n = 5 CI on ART) and IL-17A production (n = 6 HIV- and n = 6 CI on ART), respectively. The Mann Whitney p-values are indicated on the graphs. **(G)** Shown is statistical analysis of cell viability. Each symbol represents a different subject. The Mann–Whitney p-values are indicated on the graphs. Clinical parameters of subjects included in these studies are included in Table 1 (HIV- #03, 06, 07, 09, 14, 15, 19, 22) and Table 3 (CI #03, 04, 06–10, 16–18).

Mechanisms contributing to Th17 deficiency during HIV-1 infection are not fully elucidated. Here, we hypothesized that HIV-infected subjects exhibit an impaired ability to generate new Th17 cells. To test this hypothesis, we investigated the *in vitro* Th17 polarization potential of CD4^+^ T-cells expressing the naive markers CD45RA and CCR7 [49] in HIV-infected *versus* uninfected subjects. For this study, large quantities of PBMCs were collected by leukapheresis from HIV-uninfected controls (HIV-; median CD4 counts: 852 cells/μl; Table 1) and two categories of HIV-infected subjects: relatively recently infected viremics untreated (RI; median plasma viral load 14,454 HIV-RNA copies/ml; median CD4 counts 455 cells/μl; median time since infection 16 months; Table 2) and chronically infected receiving viral suppressive ART (CI on ART; plasma viral load <50 HIV-RNA copies/ml, median CD4 counts 592 cells/μl, and median time since infection 156 months; Table 3). Highly pure phenotypically naive (CD45RA^+^CCR7^+^) CD4^+^ T-cells were sorted by magnetic and then flow cytometry sorting (Additional file 1: Figure S1). Cells were cultured under Th17 polarizing conditions (TGF-β, IL-6, IL-1β, IL-23, and IL-2 recombinant cytokines and anti-IFN-γ and anti-IL-4 Abs) for 12 days (Figure 1A), using a differentiation protocol adapted from reports by other groups [33-35]. Th17-polarized cells were analyzed for the intracellular expression of IL-17A, IFN-γ, and TNF-α upon PMA/Ionomycin stimulation in the presence of Brefeldin A. The majority of Th17-polarized cells from both HIV- and CI on ART subjects expressed IL-17A in the absence of IFN-γ (IL-17A^+^IFN-γ^−^) but the presence of TNF-α (IL-17A^+^TNF-α^+^), while only very small fractions of cells were IL-17A^+^IFN-γ^+^ or IL-17A^+^TNF-α^−^ (Figure 1B). Statistical analysis demonstrated a significant decrease in the frequency of IL-17A^+^ but not IFN-γ^+^ or TNF-α^+^ cells in CI on ART compared to HIV- controls (Figure 1C). The analysis of IL-17A and IFN-γ co-expression demonstrated a significant decrease in the frequency of IL-17A^+^IFN-γ^−^ (Th17 profile), IL-17A^+^IFN-γ^+^ (Th1Th17 profile) but not IL-17A^−^IFN-γ^+^ (Th1 profile) in CI on ART compared to HIV- subjects (Figure 1D). These alterations coincided with decreased expression of RORC mRNA and a reduced production of IL-17A in culture supernatants collected at day 8 of polarization (Figure 1E-F). Alterations in the Th17 polarization potential in CI on ART subjects were associated with minor but significant alterations in the viability of Th17-polarized naive T-cells when compared to HIV- controls (Figure 1G). A similar Th17 polarization deficit was observed when experiments were performed with naive CD4^+^ T-cells from HIV^+^ RI patients (n = 5; data not shown). Finally, no significant differences where observed between CI on ART and HIV- controls regarding the Th1 polarization potential of naive T-cells (median 12.9% (n = 10) *versus* 15.45% IFN-γ^+^ cells (n = 4); Mann–Whitney p-value = 0.54; data not shown). Of note, the frequency of IL-17A^−^IFN-γ^+^ was significantly higher when naive T-cells from CI on ART subjects were cultured under Th1 (IL-2 only) *versus* Th17 conditions (12.9% *versus* 1.3%; median; n = 10; Wilcoxon p-value = 0.001; data not shown), consistent with the well-established inhibition of Th1 polarization under Th17 conditions [33-35,56].

**Table 1:** **Clinical parameters of HIV-negative subjects (HIV-)**

**Subjects**	**CD4 counts** ^**1**^	**CD8 counts** ^**1**^	**Plasma viral load** ^**2**^	**Time since infection** ^**3**^	**ART**	**ART initiation** ^**4**^	**Age** ^**5**^
**01**	1,047	430	-	-	-	-	57
**02**	1,424	605	-	-	-	-	44
**03**	732	281	-	-	-	-	51
**05**	989	582	-	-	-	-	58
**07**	1,030	340	-	-	-	-	37
**09**	998	312	-	-	-	-	51
**10**	675	208	-	-	-	-	62
**11**	*n.a.*	*n.a.*	-	-	-	-	51
**12**	*n.a.*	*n.a.*	-	-	-	-	62
**13**	*n.a.*	*n.a.*	-	-	-	-	40
**14**	733	169	-	-	-	-	45
**15**	925	272	-	-	-	-	56
**16**	665	276	-	-	-	-	44
**17**	731	308	-	-	-	-	58
**18**	1,030	383	-	-	-	-	48
**19**	634	346	-	-	-	-	68
**20**	1,115	545	-	-	-	-	65
**21**	980	448	-	-	-	-	53
**22**	620	339	-	-	-	-	49
**23**	854	492	-	-	-	-	34
**24**	1,400	678	-	-	-	-	39
**25**	918	641	-	-	-	-	30
**26**	665	276	-	-	-	-	36
**27**	521	331	-	-	-	-	21
**28**	1,030	383	-	-	-	-	49
**29**	850	412	-	-	-	-	45
**30**	918	641	-	-	-	-	46
**31**	*n.a.*	*n.a.*	-	-	-	-	24
**32**	*n.a.*	*n.a.*	-	-	-	-	48
**Median**	**918**	**364**	-				**48**

^1^, cells/μl; ^2^, HIV RNA copies per ml plasma (log_10_); ^3^, months; ART, antiretroviral therapy; ^4^, months post-infection; ^5^, years; *n.a.*, information not available.

**Table 2:** **Clinical parameters of recently HIV-infected (RI) untreated subjects**

**Subjects**	**CD4 counts** ^**1**^	**CD8 counts** ^**1**^	**Plasma viral load** ^**2**^	**Time since infection** ^**3**^	**ART**	**ART initiation** ^**4**^	**Age** ^**5**^
**RI 01**	510	491	958,435	3	No	No	43
**RI 02**	571	1,266	5,897	7	No	No	23
**RI 03**	310	350	200,363	42	No	No	48
**RI 04**	443	551	5,551	16	No	No	26
**RI 05**	345	931	318,611	34	No	No	42
**RI 06**	368	251	5,766	6	No	No	47
**RI 07**	333	628	206,719	9	No	No	34
**RI 08**	676	1,524	20,893	7	No	No	31
**RI 09**	478	495	4,898	12	No	No	30
**RI 10**	665	804	5,012	24	No	No	30
**RI 11**	397	622	14,454	23	No	No	58
**RI 12**	499	531	9,772	13	No	No	42
**RI 13**	435	1,274	36,308	24	No	No	31
**RI 14**	468	765	151,356	25	No	No	49
**RI 15**	455	365	5,370	24	No	No	40
**Median**	**455**	**622**	**14,454**	**16**	**No**	**No**	**40**

^1^, cells/μl; ^2^, HIV RNA copies per ml plasma (log_10_); ^3^, months; ART, antiretroviral therapy; ^4^, months post-infection; ^5^, years.

**Table 3:** **Clinical parameters of chronically HIV-infected subjects under long-term viral suppressive ART (CI on ART)**

**Subjects**	**CD4 counts** ^**1**^	**Nadir CD4 counts** ^**1**^	**CD8 counts** ^**1**^	**Plasma viral load** ^**2**^	**Time since infection** ^**3**^	**ART**	**ART initiation** ^**4**^	**Age** ^**5**^
**CI 01**	463	36	757	50	152	3TC-Efavirenz	105	52
**CI 02**	*n.a.*	350	*n.a.*	50	94	Kivexa-Efavirenz	24	30
**CI 03**	616	486	330	40	186	Truvada-Viracept	153	29
**CI 04**	651	219	409	40	76	Kivexa-Nevirapine	19	39
**CI 05**	358	231	283	40	155	Atripla	142	48
**CI 06**	748	20	964	40	165	Truvada-Norvir-Reyataz	134	50
**CI 07**	517	223	259	40	82	Kivexa-Sustiva	39	35
**CI 08**	269	263	282	50	*n.a.*	*n.a.*	*n.a.*	47
**CI 09**	569	299	462	50	111	Darunavir-Raltegravir	105	50
**CI 10**	391	181	620	50	165	Dalavirdine-Kivexa	34	46
**CI 11**	847	110	944	40	168	Reyataz-Kivexa	116	62
**CI 12**	498	201	531	40	213	Darunavir-Raltegravir-Intelence-Ritonavir	155	62
**CI 13**	833	486	445	40	213	Truvada-Isentress	212	31
**CI 14**	886	301	579	40	60	Truvada-Isentress	15	54
**CI 15**	824	251	900	40	58	Atripla	11	31
**CI 16**	617	24	1,272	40	157	Kivexa-Efavirenz	3	54
CI 17*	776	407	478	40	289	Atripla	231	24
**CI 18**	277	228	909	40	11	Complera	2	23
**CI 19**	459	n.a	545	40	215	*n.a.*	*n.a.*	48
**Median**	**592**	**230**	**538**	**40**	**156**	**Yes**	**105**	**47**

^1^, cells/μl; ^2^, HIV RNA copies per ml plasma (log_10_); ^3^, months; ART, antiretroviral therapy; ^4^, months post-infection; ^5^, years; *n.a.*, information not available on ART duration and regimen; *, subject infected at birth.

Together, these results provide evidence that phenotypically naive CD4^+^ T-cells from HIV^+^ subjects are impaired in their differentiation and survival potential in response to Th17- but not Th1-polarizing signals*.* This deficit is observed in CI subjects despite an efficient control of viral replication under ART.

### Phenotypically naive CD25^high^CD127^−^FoxP3^+^ and CD25^+^CD127^+^FoxP3^−^ T-cells preferentially acquire Th17 features *in vitro*

**Figure 2:**
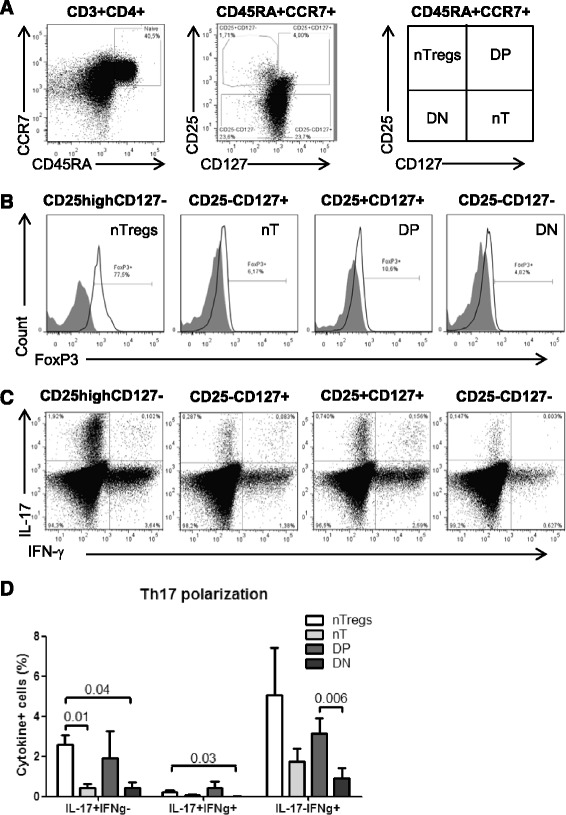
Phenotypically naive CD25^high^CD127^−^FoxP3^+^ and CD25^+^CD127^+^FoxP3^−^ T-cells preferentially differentiate into Th17 cells. PBMCs from HIV- controls were stained with a cocktail of CD3, CD4, CD45RA, CCR7, CD25 and CD127 Abs on the surface and intracellularly with FoxP3 Abs. **(A)** The differential expression of CD25 and CD127 distinguished four CD45RA^+^CCR7^+^ CD4^+^ T-cells subsets: CD25^high^CD127^−^ (nTregs), CD25^−^CD127^+^ (nT, conventional), CD25^+^CD127^+^ (DP, double positive) and CD25^−^CD127^−^ (DN, double negative). **(B)** Shown is the expression of FoxP3 on these four subsets. **(C-D)** The four CD45RA^+^CCR7^+^ T-cell subsets were sorted by flow cytometry as in Additional file 2: Figure S2, stimulated *via* CD3/CD28, and cultivated under Th17 polarizing conditions and assessed for the co-expression of IL-17A and IFN-γ as in Figure 1. Vivid staining was used to exclude dead cells. (**A-C)** Results are from one donor representative of experiments performed with cells from n = 4 donors. **(D)** Shown are statistical analysis of Th17-polarized nTregs (n = 4), nT (n = 4), DP (n = 3), and DN cells (n = 3) with differential expression of IL-17A and IFN-γ. Paired *t*-Test p-values are indicated on the graph. Clinical parameters of subjects included in these studies are included in Table 1 (HIV- # 3, 5, 6, 15).

Previous studies demonstrated that Th17 cells differentiate from naive CD4^+^ T-cells (defined as CD45RA^+^ cells) with a regulatory phenotype (nTregs: CD25^high^CD127^−^FoxP3^+^) but not from conventional naive cells (nT: CD45RA^+^CD25^−^CD127^+^FoxP3^−^) [35,50]. In addition to nTregs and nT, the differential expression of CD25, CD127, and FoxP3 identifies two other phenotypically naive CD4^+^ T-cell subsets with yet undocumented ability to undergo Th17 polarization: double positive (DP: CD25^+^CD127^+^FoxP3^−^) and double negative (DN: CD25^−^CD127^−^FoxP3^−^) subsets (Figure 2A). Here, we investigated the relative contribution of these four naive-like T-cell subsets to the pool of Th17 cells in HIV-uninfected subjects. Flow cytometry-sorted subsets (Additional file 2: Figure S2) were cultured under Th17 polarizing conditions, as in Figure 1A. Consistent with previous studies [35,50], Th17-polarized nTregs generated the highest frequency of IL-17A-expressing cells *in vitro* (Figure 2C-D). A significant fraction of cells producing IL-17A in the presence (IL-17A^+^IFN-γ^+^; Th1Th17 profile) or the absence of IFN-γ (IL-17A^+^IFN-γ^−^; Th17 profile) also originated from DP cells (Figure 2C-D). Consistent with other studies [33,34,54], a subset of Th17-polarized nTregs but also DP cells acquired Th1 features (IL-17A^−^IFN-γ^+^) when cultured under Th17 polarizing conditions (Figure 2C-D). Finally, IL-17A^+^ cells also differentiated from Th17-polarized nT and DN cells, although their frequency was significantly lower compared to nTregs (Figure 2C-D). Together, these results emphasize the phenotypic heterogeneity of phenotypically naive CD4^+^ T-cells in terms of Th17 polarization potential and identify nTregs and DP cells as being relatively enriched in Th17 lineage-committed cells.

### The frequency of nTregs and DP cells is reduced in HIV-infected subjects receiving ART

**Figure 3:**
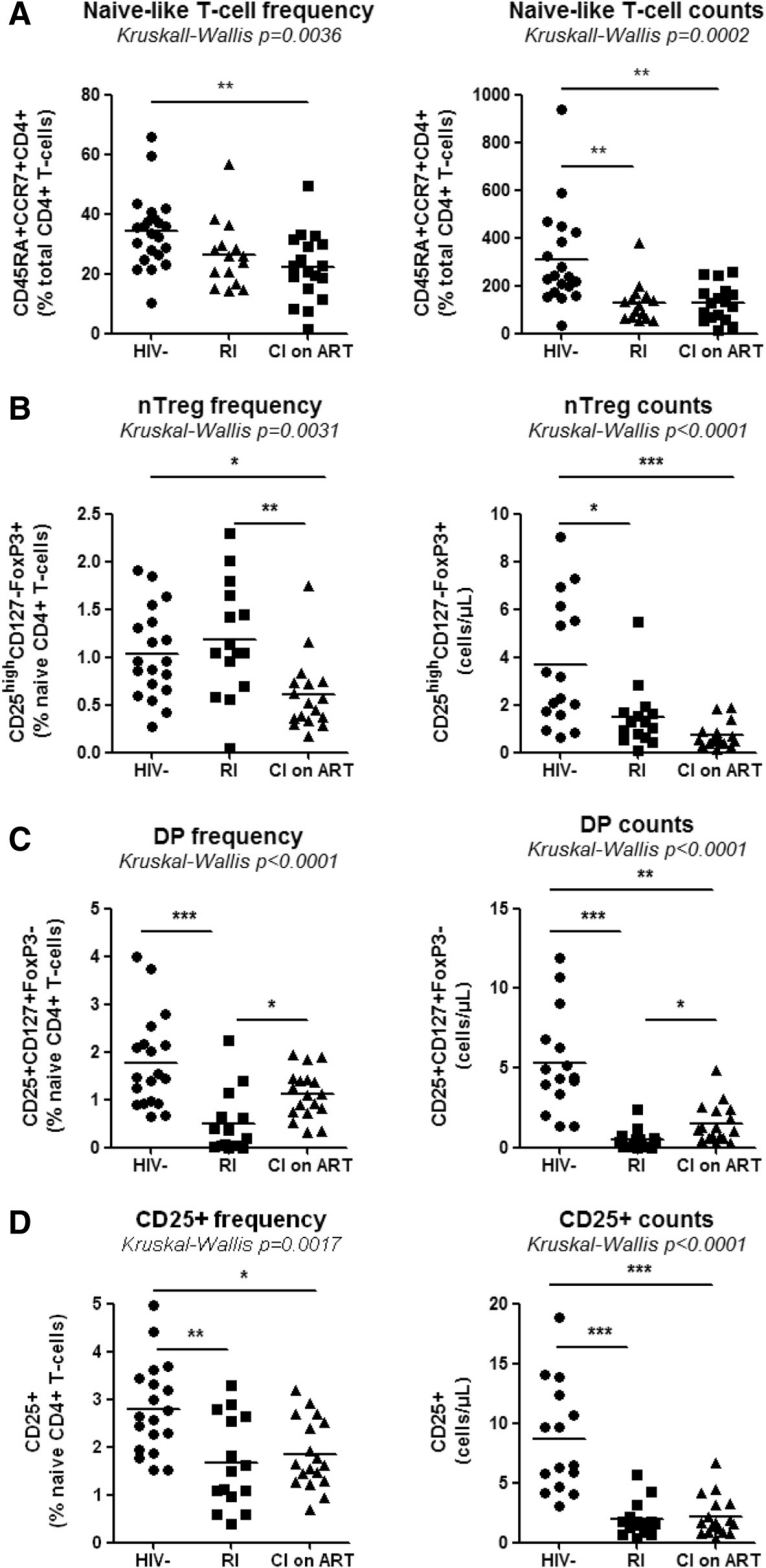
Alterations in the frequency of phenotypically naive CD4^+^ T-cell subsets in HIV-infected subjects. PBMCs from HIV- controls and HIV-infected subjects, RI and CI on ART, were analyzed for the frequency of total CD45RA^+^CCR7^+^CD4^+^ T-cells (naive-like) and with a CD25^high^CD127^−^FoxP3^+^ (nTregs) or CD25^+^CD127^+^FoxP3^−^ (DP) phenotype, together with the frequency and counts of total CD25^+^ T-cells. Cells were gated as in Figure 2. The viability dye Vivid was used to exclude dead cells. **(A-D)** Shown are the frequency (left panel) and counts (right panel) of total naive-like **(A)**, nTregs **(B)**, DP cells **(C)**, and total CD25^+^ T-cells **(D)** in the peripheral blood of HIV- (n = 19), RI (n = 15) and CI on ART (n = 18) subjects. Each symbol represents a different subject. The counts of CD45RA^+^CCR7^+^ subsets were calculated relative to their frequency and the CD4 counts. The Kruskal-Wallis and Dunns post-test p-values are indicated on the graphs (*, p < 0.05; **, p < 0.01; ***, p < 0.001). Clinical parameters of subjects used in these studies are included in Table 1(HIV- #01-03, 05, 07, 09–18, 20–23), Table 2 (RI# 1–15), and Table 3 (CI #01, 03–18).

We further investigated whether the Th17 polarization deficit in HIV-infected subjects was associated with the paucity of nTregs and DP cells. To this aim, we quantified the frequency/counts of total naive CD4^+^ T-cells and subsets expressing a nTreg and DP phenotype in the peripheral blood of CI on ART (n = 18), RI subjects (n = 15), and HIV- controls (n = 19). The absolute counts of total naive CD4^+^ T-cells, nTregs and DP cells were calculated taking into account clinical CD4 counts and the frequency of cell subsets within the CD4^+^ T-cell pool determined by flow cytometry analysis *ex vivo*. A statistically significant decrease in the frequency and/or counts of total naive CD4^+^ T-cells was observed in both CI on ART and/or RI compared to HIV- controls (Figure 3A), indicative that these HIV-infected subjects were immunologically compromised. Similar observations were made when the frequency and counts of nTregs, DP, and CD25^+^ T-cells (including both nTregs and DP cells) were compared in CI on ART and RI subjects *versus* HIV- controls (Figure 3B-D). A modest but significant increase of DP but not nTreg counts was observed in CI on ART *versus* RI subjects (Figure 3B-C), suggesting a positive effect of ART on DP cell restoration. Finally, we demonstrate a positive correlation between the yield of Th17 differentiation *in vitro* and the frequency of phenotypically naive nTregs and total CD25^+^ T-cells (Additional file 3: Figure S3). Together, these results reveal severe quantitative alterations within naive CD4^+^ T-cells, including those with nTreg and CD25^+^ phenotypes, in the peripheral blood of HIV-infected subjects despite viral suppressive ART. These alterations likely contribute to the impaired Th17 polarization observed *in vitro*.

### The paucity of nTregs and DP cells in CI on ART subjects is associated with decreased proportions of memory Th17 and Tregs

**Figure 4:**
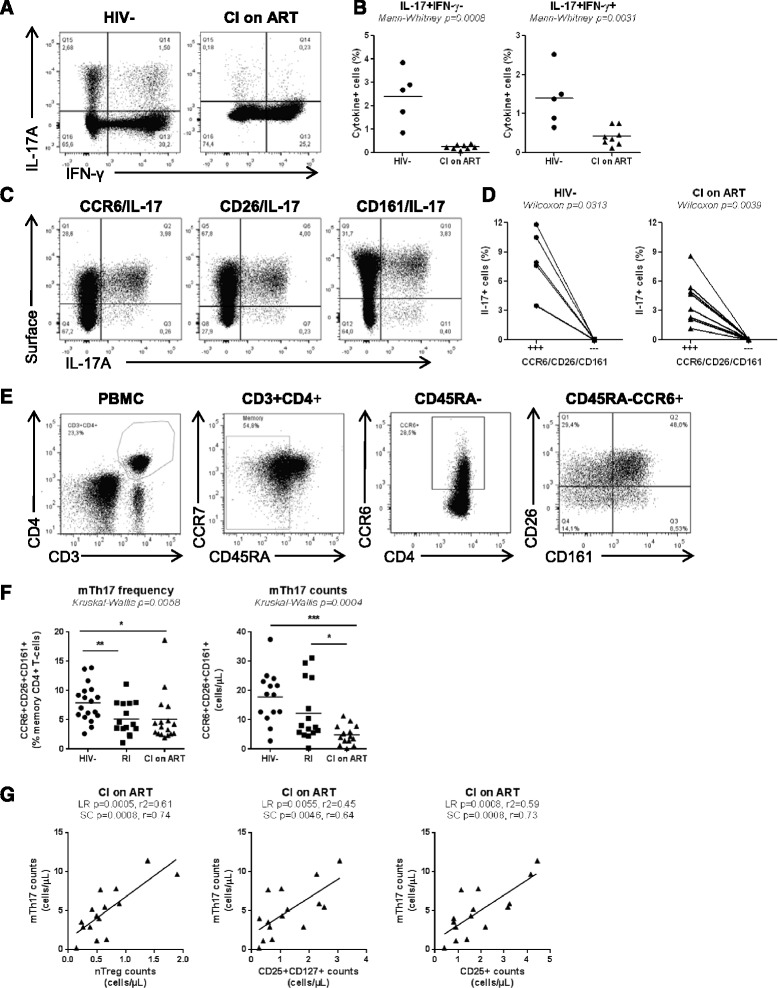
Altered frequency of memory Th17 during HIV infection in relationship with the paucity of naive-like nTreg and DP cells**. (A-D)** Memory CD4^+^ T-cells isolated by negative selection using magnetic beads (Miltenyi) were stimulated with PMA/Ionomycin and brefeldin A for 5 hours. Shown are: **(A)** representative flow cytometry dot plots illustrating IL-17A and IFN-γ expression on CD3^+^CD8^−^ T-cells from one HIV- and one CI on ART; **(B)** statistical analysis of the frequency of IL-17A^+^IFN-γ^−^ and IL-17A^+^IFN-γ^+^ cells in HIV- controls (n = 5; Table 1, HIV- #2, 10, 14, 31, 32) *versus* CI on ART subject (n = 8; Table 3, CI #3, 7–11, 14, 18); **(C)** the co-expression of CCR6, CD26 and CD161 with IL-17A in one representative HIV- control; and **(D)** statistical analysis for the frequency of IL-17A-expressing cells within CD3^+^CD8^−^ T-cells co-expressing (+++) or lacking (---) CCR6, CD26, and CD161. **(E)** Shown is the gating strategy for memory CD4^+^ T-cells (CD45RA^−^) with a mTh17 (CCR6^+^CD26^+^CD161^+^) phenotype in one representative donor. **(F)** The frequency and counts of mTh17 cells were analyzed in HIV- (n = 18; Table 1, HIV- #1-3, 5, 9–18, 20–23), RI (n = 15, Table 2, RI 11–15), and CI on ART (n = 17; Table 3, CI #1-12, 14–18) subjects. Each symbol represents a different subject. The **(B)** Mann–Whitney, **(D)** Wilcoxon, and **(F)** Kruskal-Wallis and Dunns post test p-values are indicated on the graphs (*, p < 0.05; **, p < 0.01; ***, p < 0.001). **(G)** Linear regression (LR) and Spearman correlation (SC) models were applied to determine the relationship between mTh17 cell counts and the counts of nTregs (left panel), DP (middle panel) and total naive-like CD25^+^ T-cells (right panel) in CI on ART. LR p and r^2^ and SC p and r values are indicated on the graphs. For studies in Figure 4G, subjects were identical to those included in Figure 4 F for which matched samples were available.

Previous studies documented the developmental and functional relationship between Th17 and Tregs [51,52] and profound alterations of these two lineages during progressive HIV/SIV infections [9,13]. Therefore we investigated whether the Th17 polarization deficit and the paucity of nTregs and DP cells (Figures 1 and 3; Additional file 3: Figure S3) observed in our patient cohorts (Tables 1-3) are associated with alterations in the pool of memory (CD45RA^−^) Th17 (mTh17) and Tregs (mTregs). To functionally identify mTh17 cells, we first quantified the co-expression of IL-17A and IFN-γ in memory CD4^+^ T-cells upon PMA/Ionomycin stimulation *in vitro*. No intracellular cytokines were detected in the absence of stimulation (data not shown). Results in Figure 4A-B illustrate a significantly decreased frequency of IL-17A^+^IFN-γ^−^ (Th17 profile) and IL-17A^+^IFN-γ^+^ (Th1Th17 profile) cells in CI on ART subjects (n = 8) *versus* HIV- controls (n = 5), indicative of an altered frequency of mTh17 cells. To investigate the frequency of mTh17 cells in large cohorts of HIV-infected subjects, we used the previously described Th17 surface markers CCR6, CD26, and CD161 [57]. In preliminary experiments, we validated that the majority of IL-17A-producing cells exhibit a CCR6^+^CD26^+^CD161^+^ phenotype in both uninfected controls and CI on ART subjects (Figure 4C-D). There was a positive correlation between the frequency of CCR6^+^CD26^+^CD161^+^ and that of IL-17A-producing cells (SC p = 0.04 and r = 0.9, n = 5; data not shown). Despite the fact that only a minor fraction of CCR6^+^ T-cells produce IL-17A *ex vivo*, CCR6^+^IL-17A^−^ but not CCR6^−^ cells are prone to acquire Th17 functions [58], thus justifying the use of these surface markers to predicting the frequency of mTh17 [57,59,60]. The frequency and/or counts of mTh17, identified as in Figure 4E, were significantly reduced in the peripheral blood of CI on ART and RI subjects compared to HIV- controls (Figure 4F). To establish a potential link between the paucity of nTregs and DP cells and that of mTh17 cells, SC and LR models were applied to study the correlation between the counts of these different subsets in HIV- controls and CI on ART subjects. The counts of mTh17 cells were positively correlated with the counts of nTregs, DP cells, and also total CD25^+^ T-cells in CI on ART subjects (Figure 4G). Similarly, memory Tregs (mTregs) identified as cells with a CD45RA^−^CCR7^+/−^CD25^high^CD127^−^FoxP3^+^ phenotype (Additional file 4: Figure S4A-B) were significantly depleted in frequency and/or counts in CI on ART and RI *versus* HIV- subjects (Additional file 4: Figure S4C). The counts of mTregs were positively correlated with the counts of nTregs, DP cells, and also total CD25^+^ T-cells in CI on ART (Additional file 4: Figure S4D). Thus, alterations in the frequency and counts of nTregs and DP are associated with alterations in the pools of mTh17 and mTreg in HIV-infected subjects.

### NTregs and DP cells are preferentially infected in HIV-infected subjects receiving ART

**Figure 5:**
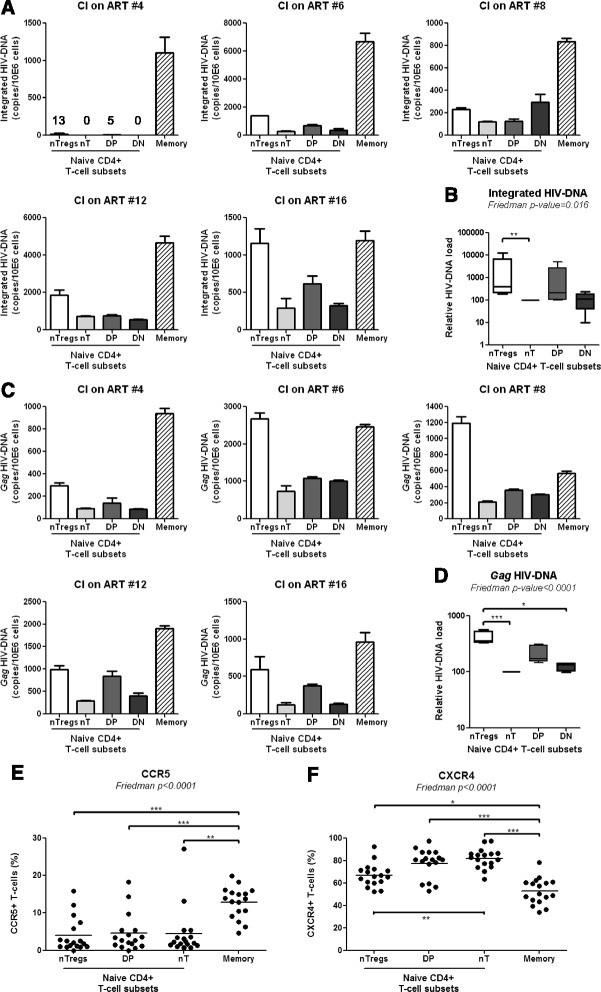
nTregs and DP cells exhibit superior permissiveness to infection. Matched total memory (CD45RA^−^) and the four subsets of CD45RA^+^CCR7^+^ CD4^+^ T-cells (nTregs, nT, DP, and DN cells) were isolated by flow cytometry from the PBMCs of CI on ART subjects as in Additional file 1 and 2: Figure S1-S2, respectively. Levels of integrated **(A)** and *Gag* **(C)** HIV-DNA were quantified by real-time PCR (equivalent of 10^5^ cells/test in triplicates) in matched samples from five CI on ART subjects (Table 3, CI #4, 6, 8, 12, 16). HIV-DNA copies were normalized to CD3 levels and expressed as HIV-DNA copies/10^6^ cells. **(A, C)** Shown are results from individuals donors. **(B, D)** Shown are statistical analysis of relative integrated and *Gag* HIV-DNA levels in naive T-cell subsets (mean ± SD, n = 4); HIV-DNA levels in conventional naive cells (nT) were considered 100%. **(E-F)** PBMCs from HIV-uninfected subjects (n = 17) were stained on the surface with CD3, CD4, CD45RA, CCR7, CD25, CD127, and CCR5 or CXCR4 Abs. The viability dye Vivid was used to exclude dead cells. The nTregs, nT, and DP cells (identified as in Figure 2A), together with total memory CD4^+^ T-cells (identified as in Figure 4E), were analyzed for the expression of CCR5 and CXCR4. Shown are the frequencies of CCR5^+^ **(E)** and CXCR4^+^ **(F)** T-cells within each subset of HIV-uninfected subjects (n = 17; Table 1, HIV- #1,2,5,10,14,15,18,20,21,23-30). The Friedman and Dunns’ post-test p-values are indicated on the graphs (*, p < 0.05; **, p < 0.01; ***, p < 0.001).

Because HIV-infection significantly contributes to CD4^+^ T-cell depletion [61-64], we investigated whether, among phenotypically naive T-cells subsets, nTreg and DP cells carry superior levels of integrated and/or non-integrated forms of HIV-DNA in CI on ART subjects. Matched nTregs, nT, DP, and DN cells, together with memory CD45RA^−^ CD4^+^ T-cells, were sorted by flow cytometry (as in Additional files 1 and 2: Figure S1-S2) from five CI on ART subjects. Integrated and *Gag* HIV-DNA was detected at different levels in T-cell subsets of 5/5 subjects (CI on ART subjects 4, 6, 8, 12, and 16; Table 3), with the infection of CD45RA^+^CCR7^+^ T-cells being inferior to that of memory CD45RA^−^ T-cells (Figure 5A and C). Then, levels of integrated and *Gag* HIV-DNA in nTregs, DP, and DN cells were analyzed relative to conventional nT cells (considered as 100%) in five CI on ART subjects (Figure 5B and D). HIV-DNA integration occurred at superior levels in nTregs *versus* nT (Figure 5B). Also, levels of *Gag* HIV-DNA were significantly higher in nTregs *versus* nT and DN cells (Figure 5D). In addition, there was a tendency for superior *Gag* HIV-DNA levels in DP *versus* nT cells from the 5/5 subjects, but results did not reach statistical significance (Figure 5D). Thus, nTregs and at a lower degree DP cells carried different forms of HIV-DNA, indicative for a superior permissiveness to HIV infection in these cells *in vivo*. To determine whether the presence of HIV-DNA in CD45RA^+^CCR7^+^ nTregs and DP cells was linked to a superior HIV entry, the expression of the HIV co-receptors CCR5 and CXCR4 was analyzed in uninfected subjects. As expected, CD45RA^+^CCR7^+^ T-cell subsets expressed lower CCR5 and higher CXCR4 levels compared to memory CD45RA^−^ T-cells, but no significant differences in CCR5 expression were observed between nTregs, DP and nT cells (Figure 5E). In contrast, nTregs but not DP cells expressed significantly lower levels of CXCR4 compared to nT cells (Figure 5F). Together, these results suggest that permissiveness to superior abortive and/or integrative HIV infection, likely regulated at post-entry levels, may represent one mechanism contributing to the depletion of nTregs and DP cells in HIV-infected subjects.

### Time of ART initiation and restoration of the Th17 deficit during chronic HIV infection

**Figure 6:**
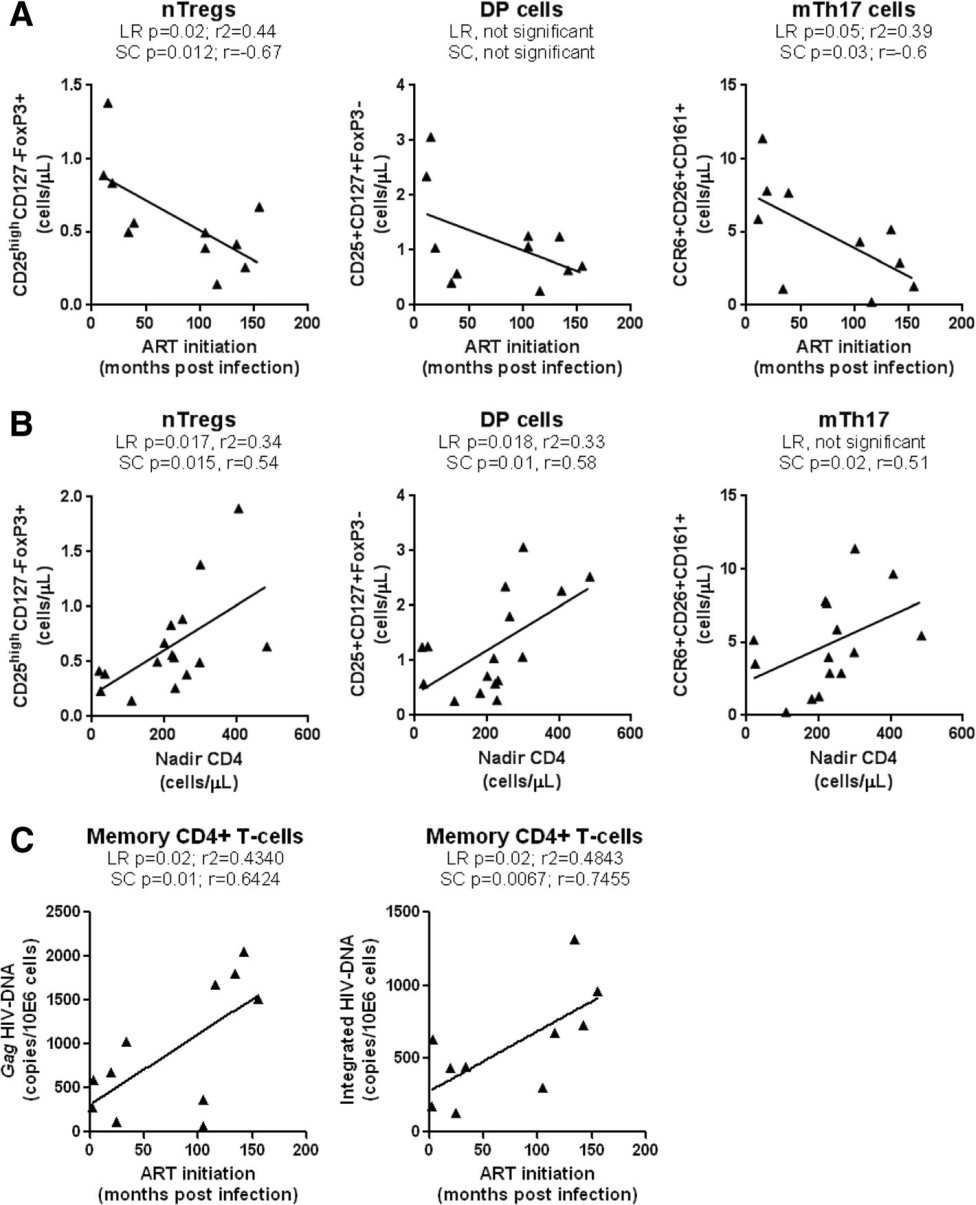
Effect of early ART initiation on the pool of nTregs, DP, and mTh17 cells. The nTregs, DP, and memory Th17 cells were phenotypically identified as in Figure 2A-B and Figure 4E, respectively. CD4 counts in CI on ART subjects are listed in Table 2. **(A)** LR and SC models were applied to determine the relationship between the time of ART initiation (months post-infection) *versus* nTreg counts (left panel), DP counts (middle panel), and Th17 counts (right panel) in CI on ART subjects (n = 11; Table 3, CI #1, 4–7, 9–12, 14–15). **(B)** LR and SC models were applied to determine the relationship between the nadir CD4 counts *versus* nTreg counts (left panel), DP counts (middle panel), and Th17 counts (right panel) in CI on ART subjects (n = 16; Table 3, CI #1, 3–12, 14–18). **(C)** Levels of *Gag* and integrated HIV-DNA were quantified by real time PCR in total memory CD4^+^ T-cells sorted by FACS as in Additional file 1: Figure S1 from CI on ART subjects (n = 11). LR and SC models were applied to determine the relationship between the levels of *Gag* (left panel) and integrated (right panel) HIV-DNA *versus* the time of ART initiation (months post-infection) in CI on ART subjects (n = 11). LR p and r^2^ values together with SC p and r values are indicated on the graphs.

Recent studies reported the benefit for early ART on immune restoration in HIV-infected subjects [65-67]. To determine whether the time of ART initiation impacts on the frequency of phenotypically naive Th17 precursors and mTh17 cells, we first studied the correlations between the time of ART initiation and the counts of nTreg and DP cells, and mTh17 cells in a group of CI on ART subjects (Table 3). Results in Figure 6A illustrate a significant negative correlation between the time of ART initiation and the counts of nTregs (LR and SC) and mTh17 (SC) but not DP cells. Of note, subjects with history of slow (*i.e.,* CI #03, 13, 17) and rapid (*i.e.,* CI #16, 18) disease progression were excluded for this analysis. The CD4^+^ T-cell counts were not correlated with the time of ART initiation (data not shown), likely because ART is highly efficient in restoring CD4 counts in the majority of subjects (median: 616 cells/μL; Table 2), regardless of the restoration of CD4^+^ T-cells heterogeneity [21]. LR and SC models were further applied to determine whether the size of the pool of nTregs and DP cells and mTh17 cells in all available CI on ART subjects can be predicted by the nadir CD4 counts before ART initiation. There was indeed a significant positive correlation between the nadir CD4 counts and the counts of nTregs, DP (LR and SC), and memory Th17 cells (SC only) (Figure 6B). Finally, an early initiation of ART was associated with reduced cell-associated *Gag* and integrated HIV-DNA levels in memory CD4^+^ T-cells (Figure 6C). These results support a model in which early ART initiation in HIV-infected subjects with relatively high nadir CD4 counts permits a better restoration of the Th17-lineage committed phenotypically naive subsets, together with mTh17 cells, likely *via* a robust control of viral replication and persistence in these cells.

**Figure 7:**
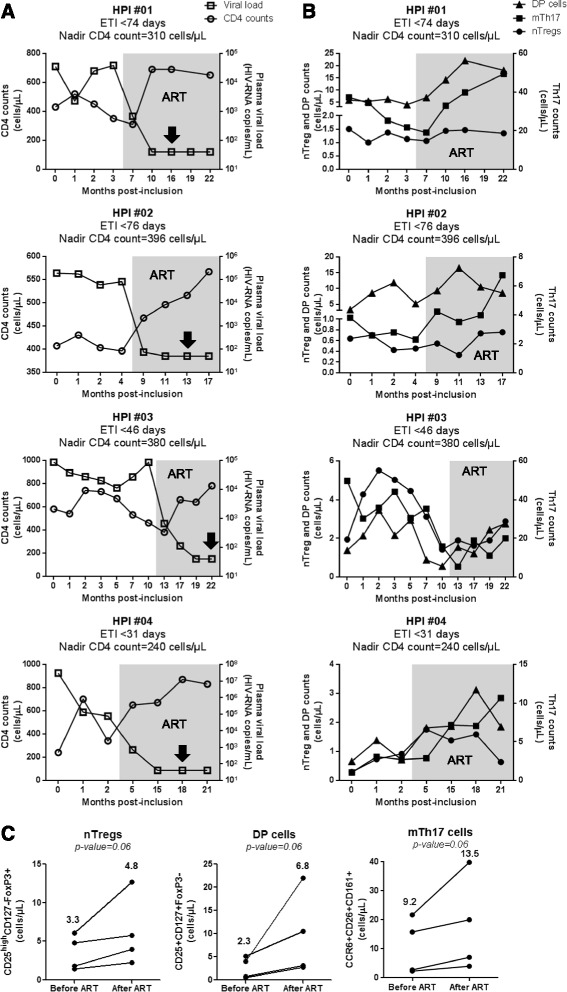
Longitudinal analysis of nTregs, DP, and mTh17 cell dynamics in HIV-infected subjects. Four HIV-infected subjects participating to the HIV Primary infection (HPI) cohort were analyzed longitudinally for the frequency and counts of nTregs, DP, and memory Th17 cells in the peripheral blood in relationship with plasma viral load and CD4 counts. Cell subsets were phenotypically identified as in Figure 2A-B and Figure 4E. The counts of nTregs, DP, and memory Th17 cells were calculated relative to their frequency and the CD4 counts. **(A)** Shown are the dynamics of CD4 counts (open circles) and plasma viral load (open squares) before and after ART initiation (grey quadrant). **(B)** Shown are the dynamics of nTreg (filled circles), DP cell (filled triangles) and memory Th17 counts (filled squares) before and after ART initiation (grey quadrant). The estimated time of infection (ETI) and the nadir CD4 count for each subject are indicated on the graphs. **(C)** Shown is statistical analyses of nTreg (left panel), DP (middle panel), and mTh17 cell counts (right panel) before and after ART. The “after ART” time points are indicated for each patient by a black arrow **(A)** and correspond to the second time point of the patient visit when plasma viral load was undetectable (<50 HIV-RNA copies/ml). Each symbol represents a different subject. Wilcoxon p-values are indicated on the graphs. Indicated in bold are median cell counts values before and after ART.

In addition to the above cross sectional studies, we also performed a longitudinal analysis in four HIV-infected subjects participating in our HIV primary infection (HPI) cohort, with an estimated time of infection (ETI) <3 months and ART initiation within the first year of infection (Table 4). We studied the dynamics of nTreg and DP cell counts, together with the counts of mTh17 cells, in relationship with total CD4^+^ T-cell counts and plasma viral load, at different time points post-inclusion before and after ART initiation. ART resulted in a rapid control of viral replication and immune restoration, as reflected by undetectable plasma viral load (<50 HIV-RNA copies/ml) and normalized CD4 counts, respectively (Figure 7A). To determine the effect of ART on nTreg, DP, and mTh17 cell restoration, these parameters were compared before ART and at a time point after ART initiation when the plasma viral load was undetectable for the second consecutive time. Despite donor-to-donor variations in the dynamic of these three subsets before and after ART, in all 4/4 donors the initiation of ART led to a modest marginally significant increase in the counts of nTregs, DP cells, mTh17 cells (Figure 7C). Together, these transversal and longitudinal studies provide evidence that the time of ART initiation impacts on the restoration/preservation of the pool of Th17-lineage committed cells.

**Table 4:** **Clinical parameters of HIV-infected subjects included in the HIV primary infection (HPI) cohort**

**Subjects**	**Visit #**	**CD4 counts** ^**1**^	**Plasma viral load** ^**2**^	**Time since infection** ^**3**^	**Time since inclusion** ^**3**^	**ART**
**HPI 01**	V1	430	35,303	2.5	0	No
	V2	520	2,301	3	1	No
ART at 8 months since infection	V3	450	24,845	3.5	2	No
	V4	350	38,395	4	3	No
	V8	310	681	9	7	Yes
	V9	690	40	12	10	Yes
	V11	690	40	21	16	Yes
	V12	-	40	21.5	19	Yes
	V13	650	40	24	22	Yes
	V14	-	-	27	25	Yes
**HPI 02**	V1	407	189,343	2.5	0	No
	V2	430	173,044	3.5	1	No
ART at 9.5 months since infection	V3	403	59,181	4	2	No
	V7	396	81,037	6.5	4	No
	V9	467	74	11.5	9	Yes
	V10	496	50	13	11	Yes
	V11	516	50	16	13	Yes
	V12	567	50	20	17	Yes
**HPI 03**	V1	580	86,627	1.5	0	No
	V2	540	37,335	2.5	1	No
ART at 11.5 months since infection	V4	740	27,348	3	2	No
	V5	730	20,076	3.5	3	No
	V7	670	11,127	5.5	5	No
	V8	530	26,574	7.5	7	No
	V9	460	85,718	10.5	10	No
	V10	380	664	13.5	13	Yes
	V11	660	113	17	17	Yes
	V12	640	40	20	19	Yes
	V13	780	40	22	22	Yes
**HPI 04**	V1	240	29,981,000	1	0	No
	V3	700	129,717	2	1	No
ART at 4.5 months since infection	V4	340	76,626	2.5	2	No
	V7	650	712	5.5	5	Yes
	V11	670	40	16	15	Yes
	V12	870	40	19	18	Yes
	V13	830	40	22	21	Yes
	V14	-	-	25	24	Yes

^1^, cells/μL; ^2^, HIV-RNA copies per mL plasma (log_10_); ^3^, months; ART, antiretroviral therapy.

## Discussion

In this manuscript, we provide evidence for the existence of a new mechanism contributing to Th17 deficiency in HIV-infected subjects. We demonstrate that phenotypically naive CD4^+^ T-cells from the peripheral blood of chronically HIV-infected subjects with undetectable plasma viral load under ART (CI on ART), as well as recently HIV-infected viremics untreated (RI), are significantly impaired in their ability to undergo Th17 polarization *in vitro.* In HIV- subjects, we reveal the existence of two phenotypically naive subsets enriched in Th17-lineage committed cells with differential expression of CD25 (IL-2 receptor alpha), CD127 (IL-7 receptor alpha) and FoxP3 (Treg master regulator of transcription): CD25^high^CD127^−^FoxP3^+^ (nTregs) and CD25^+^CD127^+^FoxP3^−^ (DP). In CI on ART, the paucity of nTregs and DP cells coincided with the dramatic depletion of memory cells producing IL-17A and/or expressing the Th17 markers CCR6, CD26, and CD161 (mTh17). Compared to conventional naive CD25^−^ T-cells (nT), nTregs and DP cells harbored superior levels of HIV-DNA, suggesting that virus-induced cell death contributes at least in part to their depletion *in vivo*. Cross-sectional and longitudinal studies revealed that early ART initiation and high nadir CD4 counts was associated with increased nTreg, DP, and mTh17 counts and reduced HIV-DNA reservoirs. All together these findings support a model in which the paucity of naive-like subsets prone to acquire Th17 functions is caused at least in part by their superior permissiveness to integrative and/or abortive infection and represents a new previously unrecognized mechanism contributing to Th17 deficiency during HIV infection.

Molecular mechanisms of Th17 deficiency during HIV/SIV infections remain a field of active investigations. Th17 deficiency in SIV-infected rhesus macaques was recently associated with superior expression of several negative regulators including the phosphatase SHP2, the suppressor of cytokine signaling 3 (SOCS3), and the protein inhibitor of activated STAT3 (PIAS3) [48]. These inhibitory molecules were previously implicated in the negative control of Th17 differentiation [68-70]. Consistent with findings in an SIV infection model, in HIV-infected subjects we report here profound alterations in the ability of peripheral blood CD4^+^ T-cells expressing the naive markers CD45RA and CCR7 [49] to survive and undergo Th17 polarization *in vitro*. The recent discovery of stem-cell memory T-cells (T_SCM_) that share CD45RA and CCR7 expression with naive T-cells [49,71,72], raises concerns on the phenotypic identification of truly naive T-cells [33-35,54,55]. Since we cannot exclude the presence of T_SCM_ in our pool of CD45RA^+^CCR7^+^ cells, in this manuscript we use the term “*phenotypically naive*” or “*naive-like*” T-cells. The Th17 polarization deficit was observed in both RI and CI on ART HIV^+^ subjects. This is consistent with previous studies by our group and others demonstrating a poor restoration of mTh17 responses in subjects receiving long-term ART [21,45,46,54]. Noteworthy, the ability of Th17- or Th1-polarized naive-like T-cells from HIV-infected subjects to produce IFN-γ was not affected, indicating that the Th17 pathway was specifically altered during HIV infection. Recent studies demonstrate that CD5 *versus* CD28 co-stimulation contributes to an enhanced ability of naive CD4^+^ T-cells to undergo Th17 polarization [56]. Also, treatment with recombinant IL-21 proved to be beneficial for the maintenance of mucosal Th17 cells in SIV-infected rhesus macaques [12,47]. Whether CD5 co-stimulation and/or IL-21 supplementation could restore the differentiation potential and improve survival of Th17-linneage committed cells in HIV-infected subjects deserves investigations.

Strong experimental evidence supports the reciprocal differentiation relationship between Th17 cells and regulatory T-cells (Tregs), with Tregs being able to convert into Th17 cells under inflammatory conditions [20,73]. Suppressive Tregs exhibit a CD25^high^CD127^−^FoxP3^+^ phenotype [74,75]. Differential expression of CD45RA and CD45RO distinguishes between naive (nTregs) and effector/memory (mTregs) Tregs [54,76]. Valmori *et al.* reported first the existence of a peripheral blood pool of Tregs (CD25^+^CTLA4^+^FoxP3^+^) exhibiting naive features (CD45RA^+^CCR7^+^CD45RO^−^CD62L^+^) and suppressive functions *in vitro,* similar to mTregs (CD45RA^−^CD45RO^+^) [77]. They also demonstrated that levels of TRECs were similarly high in nTregs and conventional naive T-cells [77]. Studies by Seddiki *et al.,* subsequently documented the existence of nTregs in different human compartments including thymus, cord and adult blood, lymph nodes and spleen [78]. Further studies by Seddiki *et al.,* identified CD127 as a new marker distinguishing Tregs from activated CD25^+^ T-cells [74]. Subsequently, several groups used this nomenclature for nTregs in humans [35,50,54,76,79-84]. It was previously reported that a fraction of human nTregs cultured under Th17 polarizing conditions differentiate into IL-17A-producing cells expressing the Th17-specific transcription factor RORγt and lacking suppressive activity *in vitro* [35,50]. In this context, we hypothesized that HIV infection was associated with alterations in the nTreg pool. We first confirmed findings by other groups [35,50] that nTregs *versus* conventional nT cells acquire IL-17A expression *in vitro*. In addition, we identified naive-like DP T-cells (CD25^+^CD127^+^FoxP3^−^) as a second source of IL-17A-producing cells. The culture under Th17-polarizing conditions of four naive-like subsets with differential expression of CD25 and CD127 generated a heterogeneous population of effector cells with Th17 (IL-17A^+^IFN-γ^−^), Th1Th17 (IL-17A^+^IFN-γ^+^), and Th1 (IL-17A^−^IFN-γ^+^) profiles, consistent with results by other groups [33,34,54]. We provide evidence that Th17-polarized nTreg and DP *versus* conventional CD25^−^CD127^+^ (nT) and CD25^−^CD127^−^ (double negative, DN) cells were enriched in IL-17A^+^IFN-γ^−^ and IL-17A^+^IFN-γ^+^ cells, indicative that fractions of nTreg and DP are committed toward the Th17 lineage. Fractions of Th17-polarized nTregs and DP also produce IFN-γ in the absence of IL-17A, thus illustrating the functional heterogeneity of nTregs and DP pools that include both Th17- and Th1-lineage committed cells.

The control of viral replication under ART is associated with the restoration of CD4 counts, but the heterogeneity and function of CD4^+^ T-cell subsets is not fully restored [21]. Here we report a dramatic decrease in the frequency/counts of nTregs and DP cells in the peripheral blood of CI on ART compared to HIV- subjects. A similar decrease was observed when the frequency of total naive-like CD25^+^ T-cells was studied. These alterations were also observed in the peripheral blood of RI viremic subjects. There was a tendency for increased frequency of DP but not nTreg counts in CI on ART *versus* RI subjects. Differences in the ability of ART to restore DP but not nTreg counts are intriguing and require further investigations.

Recent studies by Mercer *et al.* demonstrated that naive-like (CD45RO^−^) CD25^+^ T-cells differentiate into two distinct types of IL-17-producing cells: FoxP3^+^HELIOS^−^ (IL-17^+^ Tregs) and FoxP3^−^HELIOS^−^ (conventional Th17) [54]. The authors also reported a decreased frequency of IL-17^+^ Tregs in ART-treated HIV-infected subjects *versus* controls [54]. This is consistent with our findings that the pool of mTh17 and mTregs (frequency and counts) is significantly reduced in CI on ART *versus* uninfected subjects. Whether the IL-17^+^ Tregs and conventional Th17 cells described by Mercer *et al.* [54] originate from distinct Th17-lineage committed precursor pools, such as nTregs and DP cells we identified in this manuscript, remains to be investigated. Of particular importance, CD127 expression distinguishes DP from nTregs and therefore these two Th17-lineage committed pools highly likely differ in their dependency on IL-7. CD127 is preferentially expressed on memory T-cells expressing the Th17 marker CCR6 [23] and IL-7 is known to favor Th17 polarization [58]. Recent studies by our group demonstrated the positive effects of IL-7 therapy in restoring gut abnormalities in HIV-infected subjects [85]. Whether IL-7 enhances survival and Th17-polarization of DP cells remains to be investigated.

HIV-infection *per se* significantly contributes to CD4^+^ T-cell depletion [61-64]. Naive compared to memory T-cells are typically resistant to HIV infection [86,87]. However, viral entry by receptor-mediated endocytosis was documented in naive CD4^+^ T-cells, with the viral life cycle being restricted at different steps before the completion of reverse transcription and/or integration [88]. Most recently, naive CD4^+^ T-cells were documented to carry HIV-DNA in individuals receiving ART [89,90]. In some studies, abortive infection was reported to lead to T-cell depletion [62,64], while in others HIV-DNA integration was identified as a cause of virus-induced cell death [63]. Therefore we hypothesized that depletion of nTregs and DP cells was caused at least in part by their permissiveness to HIV infection. Indeed, studies by other groups demonstrated that nTregs express the HIV coreceptors CCR5 and CXCR4 and are permissive to HIV infection *in vitro* and in HIV-infected subjects [89,91,92]. Consistent with these reports, we demonstrate that nTregs compared to conventional nT-cells harbor superior levels of integrated and non-integrated HIV-DNA in CI on ART subjects. We also observed a tendency for superior integrated and non-integrated HIV-DNA levels in DP *versus* nT-cells. These differences were not linked to superior CCR5 and CXCR4 expression, suggesting that post-entry mechanisms may favor permissiveness to infection in nTregs and DP cells.

At mucosal level, SIV/HIV disease progression was linked to an altered balance between Tregs and Th17 cells to the detriment of Th17 cells [9,93]. In our study, the depletion of Th17-lineage committed nTregs and DP cells in CI on ART and/or RI subjects coincided with a significant decrease in the frequency and counts of both mTh17 and mTregs identified using well-established surface markers, together with a decreased frequency of memory CD4^+^ T-cells expressing IL-17A upon short stimulation *in vitro*. In CI on ART subjects, mTh17 counts positively correlated with the counts of naive-like nTregs, DP, and CD25^+^ T-cells, consistent with a potential differentiation relationship between these cells. Similarly, there was a positive correlation between mTreg counts and the counts of nTregs, DP, and total CD25^+^ T-cells. These results support the concept that Tregs and Th17 cells represent alternative decision fates of CD4^+^ T-cell differentiation [52,73,94]. Although the depletion of mTh17 cells during SIV/HIV infections is well documented, reports relative to nTreg/mTreg alterations are controversial (reviewed in [81]). These controversies may be explained by differences in the biological samples (peripheral blood *versus* mucosal biopsies), clinical characteristics of HIV-infected subjects included in different studies, and/or phenotypic characterization of Tregs. In line with our findings, very recent studies by Simonetta *et al.* [76] used CD25, CD127, and FoxP3 as markers for Treg identification and demonstrated that absolute numbers of both nTregs and mTregs are significantly reduced in ART-treated HIV-infected subjects [83]. Changes observed in the peripheral blood may [95] or may not [27] mirror events occurring at mucosal sites. Considering the fact that the frequency of nTregs within the naive-like T-cell pool positively correlates with the yield of Th17 polarization *in vitro*, nTregs may be used as a surrogate marker to predict Th17 alterations in HIV-infected subjects and remains to be proved.

The thymic output is reduced in HIV-infected subjects, with ART being able to restore in part these alterations [96]. The thymic function decreases with age [97] and HIV causes immune senescence and premature aging [98]. Thus, the paucity of nTregs and DP cells may be the consequence of an impaired generation of these cells by the thymus. Alterations observed in our study occurred despite insignificant age differences between HIV-infected and uninfected subjects. Whether the paucity of naive Th17 precursors is indicative of premature aging in HIV-infected subjects remains to be further investigated in larger cohorts. The expression of CD31, a surrogate marker for thymic output [90,99], was significantly higher on total CD45RA^+^CCR7^+^CD4^+^ T-cells, nTregs and DP cells in CI on ART *versus* HIV- controls (Additional file 5: Figure S5). This is consistent with reports by other groups demonstrating an effort of the thymus to compensate for lymphopenia under ART [90,100].

On a very positive note, in our cohort of CI on ART subjects we observed that the counts of nTreg and mTh17 were higher in subjects where ART was initiated early *versus* late post-infection. Moreover, we observed that nTregs, DP, and mTh17 counts were positively correlated with the nadir CD4 counts before ART. As predicted based on previous studies [87], the size of cell-associated HIV-DNA load in memory CD4^+^ T-cells was lower in subjects where treatment was initiated early and at relatively high nadir CD4 counts. Indeed, early ART initiation accelerates the decay of HIV reservoirs upon long-term treatment [67]. Finally, in a longitudinal analysis of HIV-infected subjects included in the Montreal HIV primary infection cohort we observed that ART initiation during the first year of infection tended to increase nTreg, DP, and mTh17 counts. These results indicate that the time of ART initiation and nadir CD4 counts in chronically HIV-infected subjects impacts on the levels of Th17 deficit.

## Conclusions

In this study, we *(i)* demonstrate that phenotypically naive CD4^+^ T-cells with CD25^high^CD127^−^FoxP3^+^ (nTregs) and CD25^+^CD127^+^FoxP3^−^ (DP) phenotypes are enriched in Th17-lineage committed cells in uninfected controls and *(ii)* provide evidence supporting a model in which the paucity of naive-like Th17 precursors represents a new mechanism contributing to Th17 deficiency in HIV-infected subjects. The depletion of Tregs and DP cells may be the consequence of their permissiveness to abortive and/or integrative infection. However, other causes such as alterations in the thymic output, impaired DC ability to induce Th17 polarisation [28,30], and/or limited Th17-specific growth factors (*e.g.,* IL-21 [12,47]; IL-7 [85]), cannot be excluded. Early ART initiation [16,65-67], treatment intensification with integrase inhibitors, together with other alternative therapeutic strategies aimed at Th17 restoration/preservation, will be critical for improving mucosal immunity during HIV infection.

## Methods

### Study population

HIV-infected and uninfected donors were recruited at the Montreal Chest Institute, McGill University Health Centre and Saint-Luc Hospital, Montreal, QC, Canada, through the FRQ-S/AIDS and Infectious Diseases Network (Québec, Canada). Tables 1-3 summarize immunological, virological and clinical parameters of HIV-uninfected controls (HIV-; n = 27) and HIV-infected subjects, recently infected untreated viremics (RI, n = 15) and chronically infected under ART (CI on ART; n = 19) included in transversal studies. ART included a protease inhibitor, a non-nucleoside reverse transcriptase inhibitor, and nucleoside reverse transcriptase inhibitors (Table 3). Subjects included in the HIV primo infection cohort and followed up longitudinally are described in Table 4. Plasma viral load was measured using the Amplicor HIV-1 monitor ultrasensitive method (Roche, detection threshold 50 HIV-RNA copies/ml plasma). The date of infection was estimated using clinical and laboratory data as well as patient history information. Peripheral blood mononuclear cells (PBMC) (up to 10^10^ cells) were isolated by gradient density centrifugation from leukapheresis or whole blood and frozen as previously reported [21].

### Ethics statement

This study using PBMC from HIV-infected and uninfected subjects was conducted in compliance with the principles included in the Declaration of Helsinki and received approval from the Institution Review Board of the McGill University Health Centre and CHUM-Research Centre. All subjects signed a written informed consent for their participation to the study.

### Antibodies and polychromatic flow cytometry analysis

The following fluorochrome-conjugated Abs were used for polychromatic flow cytometry analysis: CD3-Pacific Blue (UCHT1), CD4-Alexa700 (RPA-T4), CD45RA-APC-Cy7 (HI100), CCR6-PE (11A9), CCR7-PE-Cy7 (3D12), CD25-PE (M-A251), CD26-FITC (L272), CD127-AF647 (HIL-7R-M21), CD161-PE-Cy5 (DX12), IFNγ-Alexa 700 (B27), CCR5-PE (2D7), CXCR4-PE (12G5), and CD8-APC H7 (SK1) (BD Pharmingen), CD45RA-APC eFluor780 (HI100), CD56-FITC (MEM188), IL-17A-PE (eBio64DEC17), FoxP3-AF488 (PCH101), TNFα-Pacific Blue (Mab11), and IL-17A-eFluor660 (eBio64CAP17) (eBioscience), CD8-FITC (BW135/80), CD19-FITC (LT19) (Miltenyi), CCR7-PE (150503) (R&D) and CD31-BV605 (WM59) (Biolegend). Cell phenotype was analyzed by flow cytometry using the BD LSRII cytometer and BD Diva software. A viability staining Vivid (Invitrogen) was included in each staining cocktail to exclude dead cells from our analysis. FACS analysis was performed using the FlowJo software (^©^Tree Star, Inc.). For multicolor analysis, all Abs were titrated for an optimal noise/signal ratio and Abs cocktails were validated by comparing single to multiple staining. Positivity gates were placed based on fluorescence minus one (FMO), as previously described [21,101].

### Magnetic (MACS) and fluorescence activated cell sorting (FACS)

Total or memory CD4^+^ T-cells were sorted from frozen PBMCs by negative selection using magnetic beads (Miltenyi). Cell purity (typically >95%) was determined by FACS analysis upon staining with CD3-PB, CD4-Alexa700, and CD8-FITC Abs for total T-cell enrichment, together with CD45RA-APC eFluor780 and CCR7-PeCy7 Abs for memory T-cell enrichment. In some experiments (Figure 1), memory and naive subsets were sorted by FACS (BDAria II) from total CD4^+^ T-cells upon staining with CD45RA-APC-Cy7 (APC-eFluor-780) and CCR7-PE Abs and a cocktail of FITC conjugated Abs to exclude CD8^+^ T-cells (CD8), NK cells (CD56), and B cells (CD19). The sorting gates were set on CD45RA^−^ (memory) *versus* CD45RA^+^CCR7^+^ (naive T-cells) as depicted in Additional file 1: Figure S1. In other experiments (Figures 2 and 5), naive CD4^+^ T-cells subsets with differential expression of CD25 and CD127 were sorted upon staining with the cocktail above together with CD25-PE and CD127-AF647 Abs. The sorting gates were set as depicted in Additional file 2: Figure S2 using the FMOs controls, as previously described [21,101]. The viability staining Vivid (Invitrogen) was included in each staining cocktail to exclude dead cells. The post-sort quality analysis indicated that sorted subsets were in average >99% pure based on CD3, CD4 and CD45RA expression (Additional file 1 and 2: Figure S1–S2). Sorting based on CD25/CD127 expression led to a significant enrichment of naive T-cell subsets (>80%), with the exception of CD25^+^CD127^+^ which were typically >50% enriched (Additional file 2: Figure S2).

### Th17 polarization *in vitro*

Naive and memory T-cell subsets (10^6^ cells/ml) sorted by FACS were stimulated with immobilized CD3 and soluble CD28 Abs (1 μg/ml) and cultured in RPMI 1640 media supplemented with human recombinant IL-23 (50 ng/ml), TGF-β (10 ng/ml), IL-1β (10 ng/ml), and IL-6 (50 ng/ml) cytokines and neutralizing IL-4 (1 μg/ml) and IFN-γ Abs (10 μg/ml) (R&D Systems) for 12 days. Media including polarizing cytokines, Abs, and IL-2 (5 ng/ml) was refreshed at day 4 and 8 post-culture. Cells were split at day 4 and/or 8 post-culture for an optimal density 1-2x10^6^ cells/well.

### Intracellular staining for flow cytometry analysis

Cells were stimulated with PMA (50 ng/ml) and Ionomycin (1 μg/ml) in the presence of Brefeldin A (2 μg/ml) for 5 h or 17 h. The intracellular expression of IL-17A, IFN-γ and TNF-α was quantified by flow cytometry (BD LSRII) upon staining with appropriate Abs using the Cytofix/Cytoperm kit (BD Biosciences) according to the manufacturer’s protocols. The intracellular expression of FoxP3 was quantified using the Anti-Human Foxp3 Staining Set Alexa Fluor® 488 (eBioscience) according to the manufacturer’s protocol.

### ELISA quantification of IL-17A production

IL-17A levels in cell culture supernatants were quantified by a specific ELISA assay (eBiosciences) according to the manufacturer’s protocol.

### Quantitative SYBR green real-time RT-PCR

Total RNA was isolated using RNeasy kit (Qiagen). The quality (260/280 ratio) and quantity of RNA collected were measured by a Pearl nanophotometer (Implen, Munich, Germany). One step SYBR Green real-time RT-PCR (Qiagen) was carried out in a LightCycler 480 II (Roche) according to the manufacturer’s recommendations. The quantification of RORC mRNA relative to the 28S rRNA levels was performed as we previously described [21]. Each RT-PCR reaction was performed in triplicates.

### Real-time PCR quantification of *Gag* and integrated HIV-DNA

The quantification of *Gag* and integrated HIV-DNA was performed as we previously described [21,87]. Briefly, cells were digested in a proteinase K buffer (Invitrogen), and 10^5^ cells/15 μl lysate were used *per* amplification. Integrated HIV-DNA was amplified first (12 cycles) using two outward-facing *Alu* primers and one HIV LTR primer tagged with a lambda sequence; the CD3 gene was amplified in the same reaction. The HIV and CD3 amplicons were then amplified in separate reactions (Light Cycler 480, Roche Diagnostics). The HIV-DNA was amplified using a lambda-specific primer and an inner LTR primer in the presence of two fluorescent probes specific for HIV LTR. The CD3 DNA was amplified using inner primers and two fluorescent probes specific for CD3. Similarly, total HIV-DNA was quantified using two sets of outward and inner *Gag* primers [21,87]. The sensitivity of the nested real-time PCR was 3 copies Gag and integrated HIV-DNA, as previously described [21,87]. Amplification reactions were carried out with Light Cycler 480 Probe Master Mix (Roche) and Taq Polymerase (Invitrogen). The ACH2 cells carrying one copy of integrated HIV-DNA per cell (NIAIDS reagent program) were used as standard curve.

### Statistical analysis

All statistical analyses were performed using the GraphPad Prism 5 software and details are included in Figure legends.

## Additional files

Additional file 1: Figure S1. Flow cytometry sorting of CD4^+^ T-cell subsets. Total CD4^+^ T-cells were isolated from PBMCs by negative selection using magnetic beads (Miltenyi). Cells were stained with a cocktail of CD45RA, CCR7, CD8, CD19, and CD56 Abs. The viability marker Vivid was used to exclude dead cells. Naive-like (CD45RA^+^CCR7^+^ phenotype) and memory (CD45RA^−^CCR7^+/−^ phenotype) CD4^+^ T-cells lacking CD8, CD19, and CD56 expression were sorted by flow cytometry. Shown is the phenotype of cells before and after cell sorting by MACS and then FACS. Results were generated with cells from one donor representative of experiments performed with cells from different HIV-infected (n = 10) and uninfected donors (n = 8). The purity of MACS and FACS sorted T-cells is indicated in the Figure as the % of cells exhibiting a specific phenotype. Additional file 2: Figure S2. Flow cytometry sorting of phenotypically naive CD4^+^ T-cells with differential expression of CD25 and CD127. Total CD4^+^ T-cells were isolated from PBMCs by negative selection using magnetic beads (Miltenyi). Cells were stained with a cocktail of CD3, CD4, CD45RA, CCR7, CD25, and CD127 Abs and the viability dye Vivid. Viable (Vivid^−^) naive-like (CD45RA^+^CCR7^+^) CD4^+^ T-cells with a CD25^+^CD127^−^ (nTregs), CD25^−^CD127^+^ (conventional nT), CD25^+^CD127^+^ (DP, double positive), and CD25^−^CD127^−^ (DN, double negative) phenotype were sorted by flow cytometry. Shown are results from one donor representative of experiments performed with cells from n = 3 HIV- controls and n = 5 CI on ART subjects. The purity of MACS and FACS sorted T-cells is indicated on the Figure as the % of cells exhibiting a specific phenotype. Additional file 3: Figure S3 The frequency of nTregs and CD25^+^ T-cells predicts the yield of Th17 polarization *in vitro*. Spearman correlation (SC) and linear regression models (LC) were applied to determine the relationship between the frequency of nTregs, DP, and CD25^+^ T-cells *ex vivo* (as described in Figure 3) and the ability of phenotypically naive CD4^+^ T-cells to acquire Th17 functions upon polarization *in vitro* (as described in Figure 1). Results are from matched HIV- controls (n = 7; filled circles) and CI on ART subjects (n = 10; filled triangles). Subjects were identical to those included in Figure 1C-G for which matched samples were available. Additional file 4: Figure S4 Altered frequency of mTregs during HIV infection in relationship with the paucity of nTreg and DP cells. PBMCs from HIV-infected, RI and CI on ART, and uninfected subjects were stained with a cocktail of CD3, CD4, CD45RA, CCR7, CD25, and CD127 Abs on the surface and with FoxP3 Abs intracellular, and analyzed by flow cytometry. CD45RA^+^CCR7^+^ nTregs and DP cells were identified as in Figure 2A. (A) Shown is the gating strategy for the identification of memory (CD45RA^−^) T-cell subsets with a CD25^high^CD127^−^ surface phenotype specific for memory Tregs (mTregs). (B) Shown is the intracellular expression of FoxP3 in CD25^high^CD127^−^ *versus* CD25^−^CD127^+^ subsets. (A-B) Results are from one donor representative of results generated with cells from n = 18 CI on ART and n = 18 uninfected subjects. **(C)** The frequency (left panel) and counts (right panel) of mTregs were analyzed in HIV- (n = 18) and CI on ART (n = 18) subjects. Each symbol represents a different subject. The Kruskal-Wallis and Dunns post test p-values are indicated on the graphs (*, p < 0.05; **, p < 0.01; ***, p < 0.001). (D) Linear regression (LR) and Spearman correlation (SC) models were applied to determine the relationship between mTreg counts and the counts of nTregs (left panel), DP cells (middle panel) and total naive-like CD25^+^ T-cells (right panel) in CI on ART subjects. LR p and r^2^ values together with SC p and r values are indicated on the graphs. Clinical parameters of subjects used for studies in Additional file 4: Figure S4C are included in Table 1 (HIV- #1-3, 5, 7, 9–18, 20–23), Table 2 (RI# 1–15), and Table 3 (CI #1, 3–18). For studies in Additional file 4: Figure S4D, subjects were identical to those included in 4C, except those for which CD4 counts were not available. Additional file 5: Figure S5 Expression of CD31 on nTregs and DP cells in CI on ART *versus* uninfected subjects. PBMCs from HIV-uninfected and HIV-infected CI on ART subjects were stained on the surface with CD3, CD4, CD45RA, CCR7, CD25, CD127 and CD31 Abs. The viability dye Vivid was used to exclude dead cells. **(A)** Shown are the frequencies of CD31^+^ cells on total naive-like (CD45RA^+^CCR7^+^) CD4^+^ T-cells, nTregs, and DP cells from HIV- controls *versus* CI on ART subjects. The Mann–Whitney p-values are indicated on the graphs. **(**B) Shown is the analysis of the relationship between the counts of total naive-like CD4^+^ T-cells, nTregs, and DP cells and the frequency of CD31^+^ cells in each subset. LR p and r^2^ values together with SC p and r values are indicated on the graphs. Clinical parameters of subjects used for studies in this figure are included in Table 1 (HIV- #1, 2, 5, 10, 14, 15, 18, 20, 21, 23–30) and 1C (CI #1-3, 6–19).
