# Supplementary figures and images for: Impaired Th17 polarization of phenotypically naive CD4+ T-cells during chronic HIV-1 infection and potential restoration with early ART
Source: Retrovirology. 2015 Apr 30;12:38. doi: 10.1186/s12977-015-0164-6 (PMC4438463; doi:10.1186/s12977-015-0164-6)

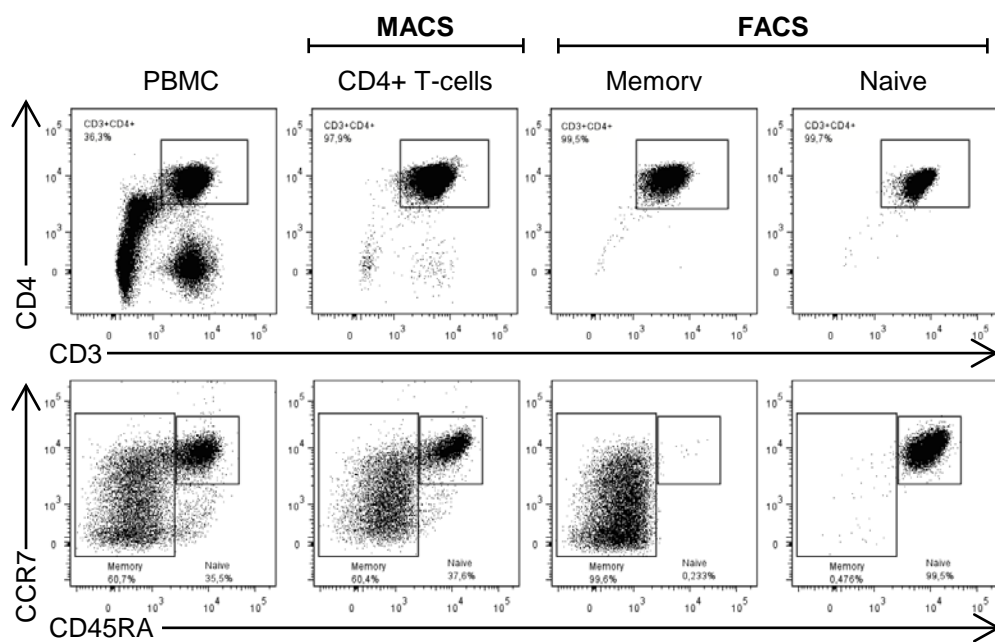

Supplement: Additional file 1: Figure S1. — Flow cytometry sorting of CD4+ T-cell subsets. Total CD4+ T-cells were isolated from PBMCs by negative selection using magnetic beads (Miltenyi). Cells were stained with a cocktail of CD45RA, CCR7, CD8, CD19, and CD56 Abs. The viability marker Vivid was used to exclude dead cells. Naive-like (CD45RA+CCR7+ phenotype) and memory (CD45RA−CCR7+/− phenotype) CD4+ T-cells lacking CD8, CD19, and CD56 expression were sorted by flow cytometry. Shown is the phenotype of cells before and after cell sorting by MACS and then FACS. Results were generated with cells from one donor representative of experiments performed with cells from different HIV-infected (n = 10) and uninfected donors (n = 8). The purity of MACS and FACS sorted T-cells is indicated in the Figure as the % of cells exhibiting a specific phenotype. [file 12977_2015_164_MOESM1_ESM.pdf]

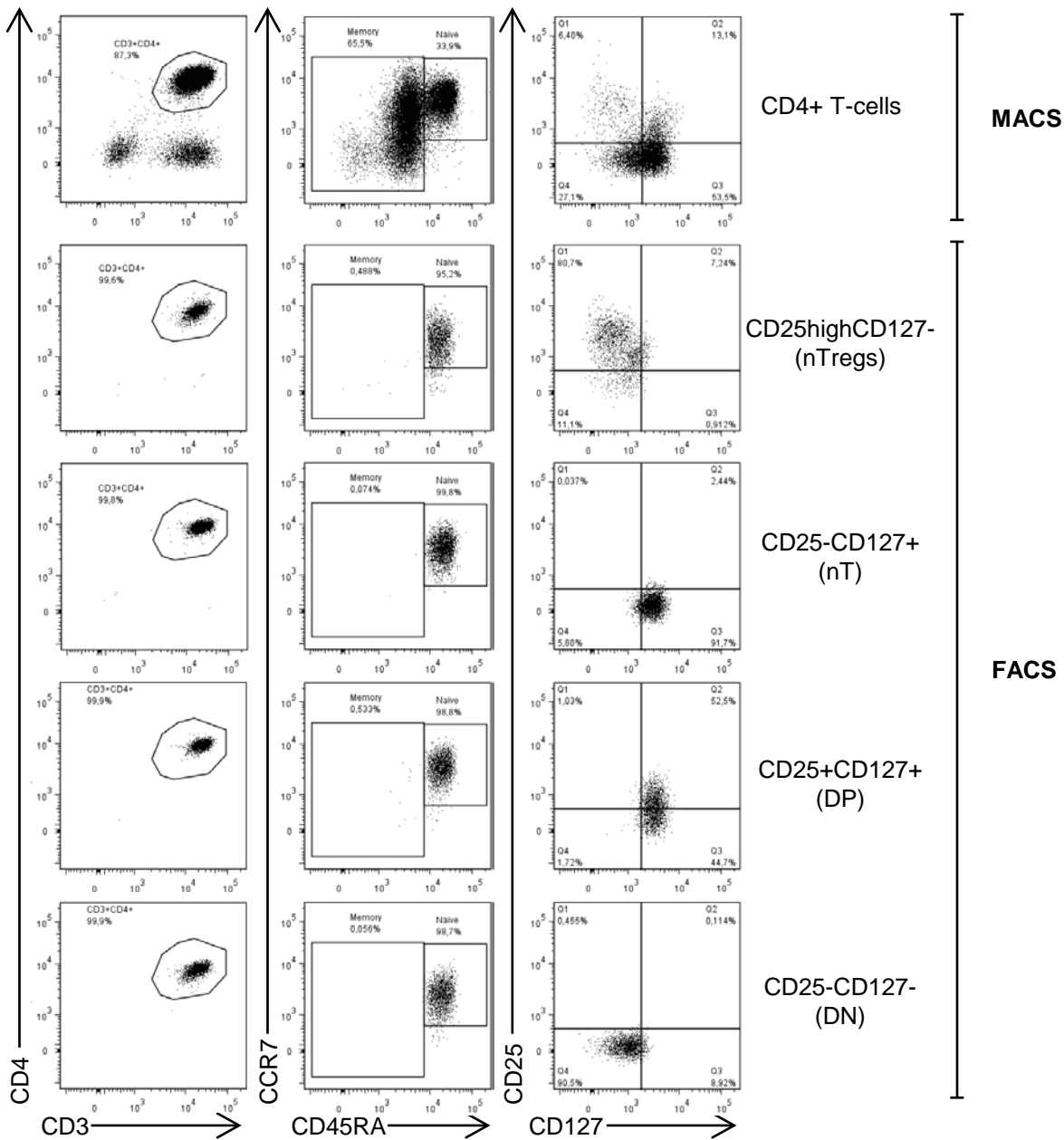

Supplement: Additional file 2: Figure S2. — Flow cytometry sorting of phenotypically naive CD4+ T-cells with differential expression of CD25 and CD127. Total CD4+ T-cells were isolated from PBMCs by negative selection using magnetic beads (Miltenyi). Cells were stained with a cocktail of CD3, CD4, CD45RA, CCR7, CD25, and CD127 Abs and the viability dye Vivid. Viable (Vivid−) naive-like (CD45RA+CCR7+) CD4+ T-cells with a CD25+CD127− (nTregs), CD25−CD127+ (conventional nT), CD25+CD127+ (DP, double positive), and CD25−CD127− (DN, double negative) phenotype were sorted by flow cytometry. Shown are results from one donor representative of experiments performed with cells from n = 3 HIV- controls and n = 5 CI on ART subjects. The purity of MACS and FACS sorted T-cells is indicated on the Figure as the % of cells exhibiting a specific phenotype. [file 12977_2015_164_MOESM2_ESM.pdf]

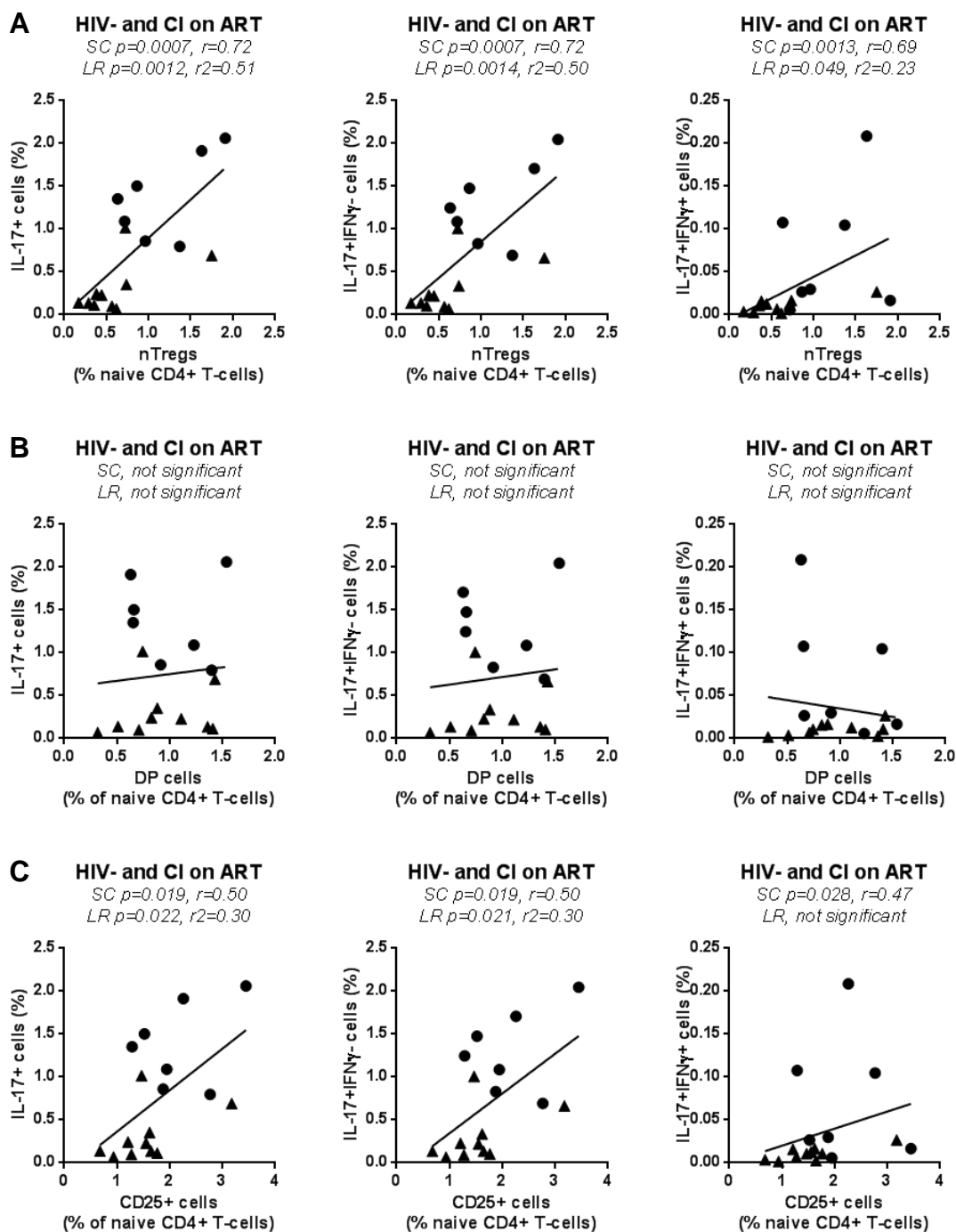

Supplement: Additional file 3: Figure S3 — The frequency of nTregs and CD25+ T-cells predicts the yield of Th17 polarization in vitro. Spearman correlation (SC) and linear regression models (LC) were applied to determine the relationship between the frequency of nTregs, DP, and CD25+ T-cells ex vivo (as described in Figure 3) and the ability of phenotypically naive CD4+ T-cells to acquire Th17 functions upon polarization in vitro (as described in Figure 1). Results are from matched HIV- controls (n = 7; filled circles) and CI on ART subjects (n = 10; filled triangles). Subjects were identical to those included in Figure 1C-G for which matched samples were available. [file 12977_2015_164_MOESM3_ESM.pdf]

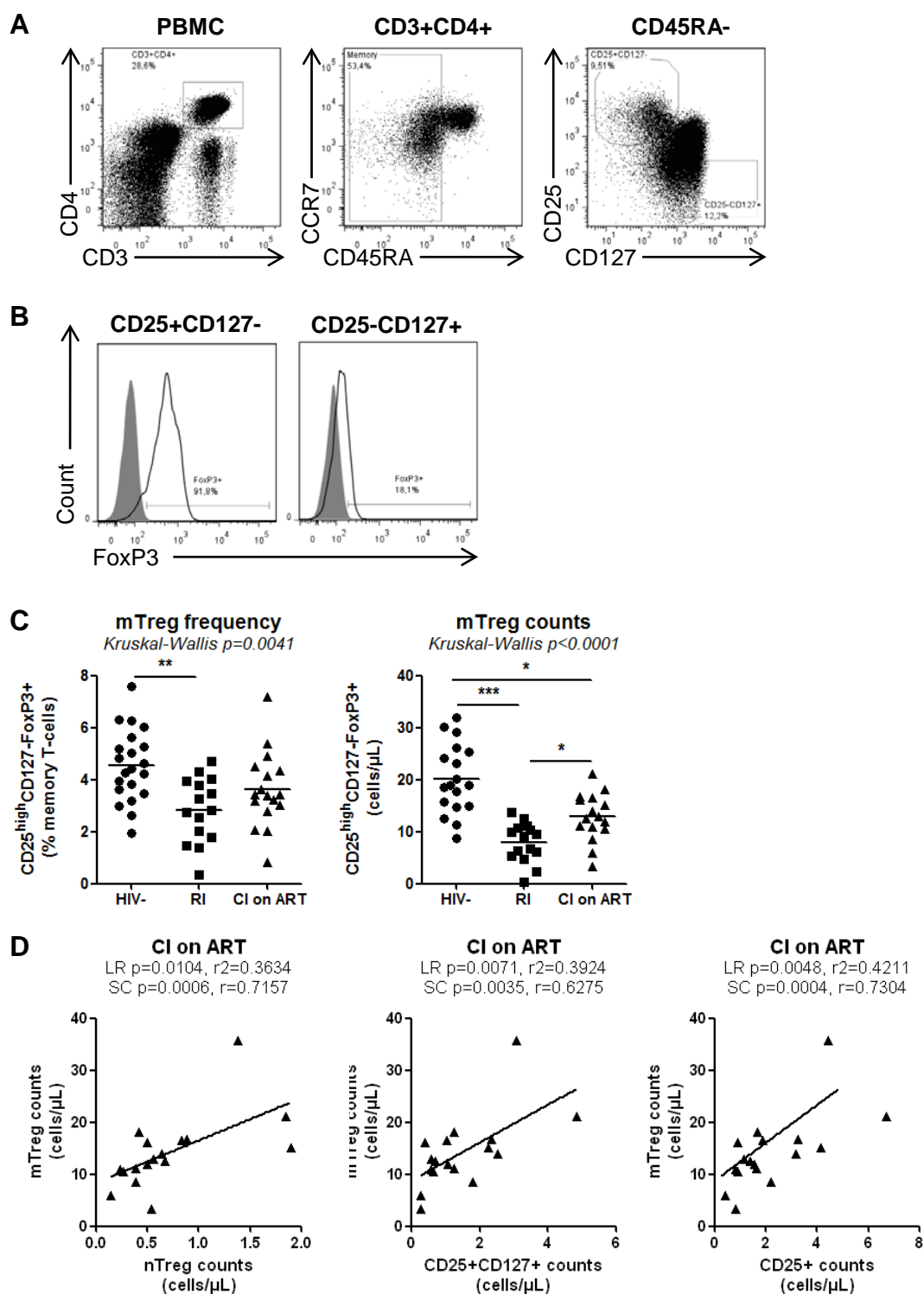

Supplement: Additional file 4: Figure S4 — Altered frequency of mTregs during HIV infection in relationship with the paucity of nTreg and DP cells. PBMCs from HIV-infected, RI and CI on ART, and uninfected subjects were stained with a cocktail of CD3, CD4, CD45RA, CCR7, CD25, and CD127 Abs on the surface and with FoxP3 Abs intracellular, and analyzed by flow cytometry. CD45RA+CCR7+ nTregs and DP cells were identified as in Figure 2A. (A) Shown is the gating strategy for the identification of memory (CD45RA−) T-cell subsets with a CD25highCD127− surface phenotype specific for memory Tregs (mTregs). (B) Shown is the intracellular expression of FoxP3 in CD25highCD127− versus CD25−CD127+ subsets. (A-B) Results are from one donor representative of results generated with cells from n = 18 CI on ART and n = 18 uninfected subjects. (C) The frequency (left panel) and counts (right panel) of mTregs were analyzed in HIV- (n = 18) and CI on ART (n = 18) subjects. Each symbol represents a different subject. The Kruskal-Wallis and Dunns post test p-values are indicated on the graphs (*, p < 0.05; **, p < 0.01; ***, p < 0.001). (D) Linear regression (LR) and Spearman correlation (SC) models were applied to determine the relationship between mTreg counts and the counts of nTregs (left panel), DP cells (middle panel) and total naive-like CD25+ T-cells (right panel) in CI on ART subjects. LR p and r2 values together with SC p and r values are indicated on the graphs. Clinical parameters of subjects used for studies in Additional file 4: Figure S4C are included in Table 1 (HIV- #1-3, 5, 7, 9–18, 20–23), Table 2 (RI# 1–15), and Table 3 (CI #1, 3–18). For studies in Additional file 4: Figure S4D, subjects were identical to those included in 4C, except those for which CD4 counts were not available. [file 12977_2015_164_MOESM4_ESM.pdf]

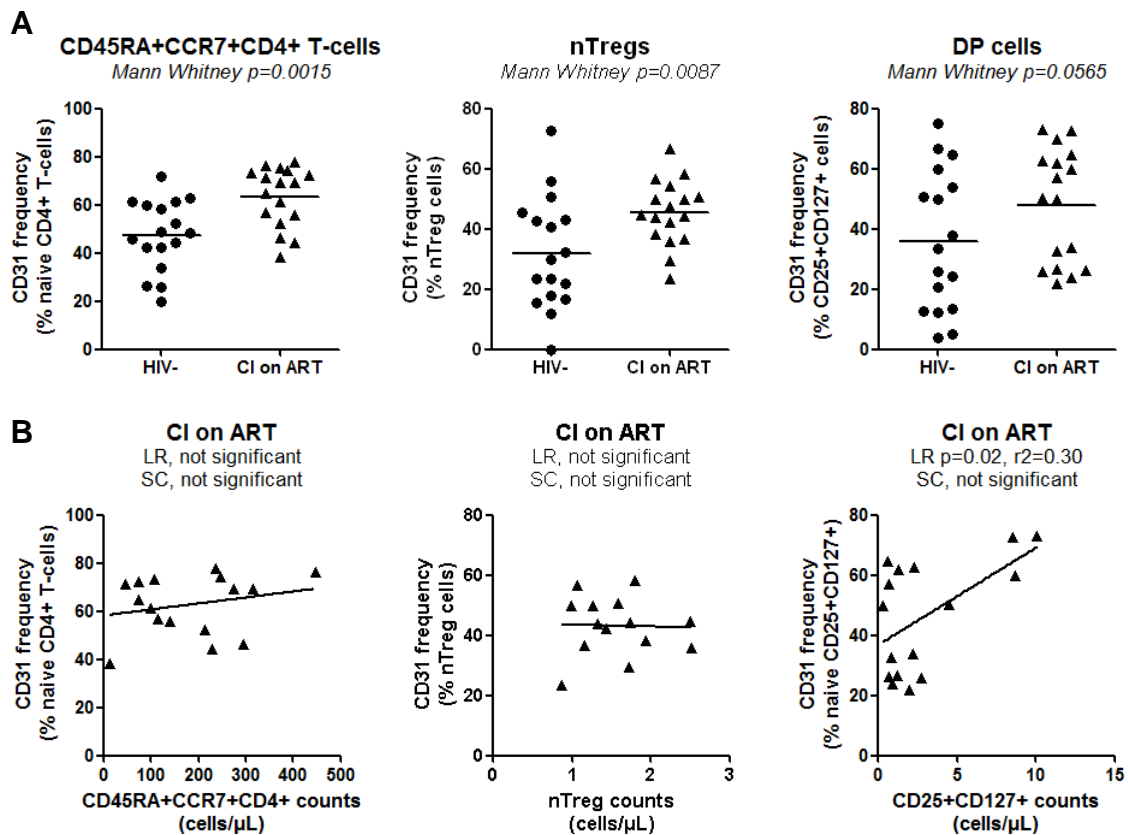

Supplement: Additional file 5: Figure S5 — Expression of CD31 on nTregs and DP cells in CI on ART versus uninfected subjects. PBMCs from HIV-uninfected and HIV-infected CI on ART subjects were stained on the surface with CD3, CD4, CD45RA, CCR7, CD25, CD127 and CD31 Abs. The viability dye Vivid was used to exclude dead cells. (A) Shown are the frequencies of CD31+ cells on total naive-like (CD45RA+CCR7+) CD4+ T-cells, nTregs, and DP cells from HIV- controls versus CI on ART subjects. The Mann–Whitney p-values are indicated on the graphs. (B) Shown is the analysis of the relationship between the counts of total naive-like CD4+ T-cells, nTregs, and DP cells and the frequency of CD31+ cells in each subset. LR p and r2 values together with SC p and r values are indicated on the graphs. Clinical parameters of subjects used for studies in this figure are included in Table 1 (HIV- #1, 2, 5, 10, 14, 15, 18, 20, 21, 23–30) and 1C (CI #1-3, 6–19). [file 12977_2015_164_MOESM5_ESM.pdf]
